# Utilizing virus genomic surveillance to predict vaccine effectiveness

**DOI:** 10.1371/journal.pcbi.1014329

**Published:** 2026-05-26

**Authors:** Jiye Kwon, Ke Li, Joshua L. Warren, Sameer Pandya, Anne M. Hahn, Yale SARS-CoV-2 Genomic Surveillance Initiative, Virginia E. Pitzer, Daniel M. Weinberger, Nathan D. Grubaugh

**Affiliations:** 1 Department of Epidemiology of Microbial Diseases, Yale School of Public Health, New Haven, Connecticut, United States of America; 2 Public Health Modeling Unit, Yale School of Public Health, New Haven, Connecticut, United States of America; 3 Department of Biostatistics, Yale School of Public Health, New Haven, Connecticut, United States of America; 4 Department of Laboratory Medicine, Yale School of Medicine, New Haven, Connecticut, United States of America; 5 Yale School of Medicine Biorepository, Yale University, New Haven, Connecticut, United States of America; 6 Department of Microbiology and Immunology, University of Melbourne, at the Peter Doherty Institute for Infection and Immunity, Melbourne, Victoria, Australia; 7 Department of Ecology and Evolutionary Biology, Yale University, New Haven, Connecticut, United States of America; University of California San Diego, UNITED STATES OF AMERICA

## Abstract

**Background:**

Since the development of the first vaccines targeting the original SARS-CoV-2 virus sequence in 2020, mRNA-based vaccines have been updated three times: targeting Omicron BA.4/BA.5 in 2022, the XBB lineage in 2023, and the KP.2 variant in 2024. While genomic surveillance has advanced our understanding of pathogen diversity, gaps remain in incorporating genomic information to evaluate vaccine effectiveness (VE) against emerging variants. This study aims to characterize the relationship between VE and sequence-based genetic distance, to establish a framework for predicting near real-time changes in the level of vaccine protection from virus surveillance data.

**Methods:**

We analyzed 10,156 whole genome sequences of SARS-CoV-2 cases from Connecticut, USA, between April 2021 to July 2024. We first assessed how genetic distance, specifically the number of amino acid substitutions in the spike gene between COVID-19 case sequences and the mRNA vaccine formulation sequence(s), correlates with vaccine protection levels. Incorporating data from over 1 million test-negative controls, we developed a Bayesian time-varying model with autoregressive terms to assess VE at a weekly level. The analysis was adjusted for ZIP-code-level income, age, sex, and prior vaccine doses received. We then employed a random effects meta-regression to explore the relationship between VE and amino acid distance over time. Finally, we used the meta-regression model to estimate potential vaccine protection against emerging variants.

**Findings:**

We found that spike gene amino acid distance showed a negative correlation with VE over time. Stepwise increases in amino acid distance aligned with sharp VE declines during variant emergence, while accumulation of within-variant changes was also associated with gradual VE decline. Each 10 amino acid increase in distance in the spike gene corresponds to a predicted 15.4% (95% credible intervals (CrI): –2.0%, 34.6%) reduction in VE. For the 2023/24 updated vaccine, spike distance rose from 12.25 to 30.23, predicting a 43.4% (95% CrI: –5.7%, 90.1%) drop in VE using sequence information alone.

**Conclusion:**

Our framework quantifies how the emergence of new variants is expected to affect VE for SARS-CoV-2. By quantifying the relationship between amino acid substitutions and time-varying VE, we leverage intrinsic pathogen features, such as spike amino acid distance, to inform future vaccine updates using genomic sequences. As genomic surveillance data becomes more widely available across pathogens, this framework can serve as a near-real time surveillance tool to infer population-level protection and offers valuable insights for vaccine update decisions.

## Author summary

Rapidly evolving viruses present a significant challenge for maintaining vaccine effectiveness (VE). We developed a model to estimate the impact of antigenic variation on VE in near real-time, applying it to SARS-CoV-2. We analyzed 10,156 genomes from Connecticut and over one million control records to quantify the relationship between VE and genetic distance from mRNA vaccine strains. There was an inverse correlation: every 10 amino acid substitutions corresponded to a 15.4% (95% credible intervals (CrI): –2.0%, 34.6%) reduction in VE. This corresponds to a 43.4% (95% CrI: –5.7%, 90.1%) drop in VE for the 2023/24 vaccine following the emergence of JN.1 variants. By linking amino acid substitutions to VE, this approach leverages genomic surveillance to monitor population-level protection and inform timely vaccine updates.

## Introduction

The antigenic evolution of pathogens, particularly RNA viruses, presents a persistent challenge for disease control and remains a major contributor to global disease burden [1–3]. As viruses evolve to escape population immunity, they can repeatedly infect individuals, potentially fueling recurrent epidemics [2,4]. Viral evolution and epidemiology are deeply interconnected, with transmission both driving and being shaped by viral adaptation [2,5]. Achieving population-level immunity through vaccination is a critical public health strategy [6]. However, when immunity is strain-specific, it leads to opportunities for viral adaptation through immune escape [7–9]. The resulting decline in population-level vaccine effectiveness (VE) necessitates periodic updates for certain vaccines. Influenza viruses serve as a well-established example, with global surveillance data guiding regular vaccine updates to maintain protection [10,11]. More recently, the rapid evolution of SARS-CoV-2 highlights yet another need for frequent vaccine updates. However, COVID-19 vaccine updates have been undertaken annually and largely *ad hoc*. This reflects a broader issue. As vaccines are developed for these and other fast-evolving viruses, determining when and how to update them—and what data should inform these decisions—remains a critical challenge. Addressing this gap is crucial for maintaining VE and optimizing long-term disease control.

In the United States (US), the Advisory Committee on Immunization Practices (ACIP) issues recommendations for the use of updated COVID-19 mRNA vaccines, guided by a rigorous evaluation of VE data in conjunction with clinical, epidemiological, safety, and economic evidence [12]. The timeline for the 2024/25 COVID-19 vaccine update illustrates this process [13]. The 2023/24 XBB.1.5 mRNA vaccine became available in September 2023 [14]. In February 2024, ACIP reviewed VE data from the VISION and IVY networks [15], supplemented by genomic surveillance on SARS-CoV-2 lineage proportions (e.g., XBB, JN.1) from the Centers for Disease Control and Prevention (CDC) [15]. In June 2024, ACIP unanimously recommended the 2024/25 KP.2 mRNA vaccine formula for all individuals above 6 months old [12]; the Food and Drug Administration (FDA) gave full approval to the updated mRNA vaccines from Moderna and Pfizer in August 2024 [16]; and the vaccines became available to the public by October 2024 [17]. However, important gaps remain. Reduced VE of the XBB.1.5 vaccine against the JN.1 was identified in February 2024—months after JN.1 began circulating. Yet, the updated JN.1 lineage-based formula (e.g., KP.2) was not available until October. By this time, KP.3.1.1 had become the dominant lineage, and XEC—a recombinant of two JN.1 lineages—was on the rise [18], eroding the benefits of the updated vaccine. This delay underscores the need for a more adaptive vaccine update process. Moreover, proposed FDA guidelines requiring randomized controlled trials to license future COVID-19 updates for use in those under 65 years of age in the US [19] further underscore the need for evaluation tools that can assess both when to update the vaccine and the continued effectiveness of previous formulations when updated vaccines are not readily available to all age groups.

Routine genomic surveillance data offer a crucial opportunity to bridge this gap by enabling timely detection of emerging variants with the potential for immune escape. Integrating genomic data with disease surveillance enhances our understanding of infection dynamics, identifies key drivers of change, and informs tailored intervention strategies, including vaccination [20]. A genomics-informed early warning system hinges on linking four key data sources: virus sequence data, epidemiological data, demographic data, and immunization data [21]. In this study, we leverage these sources of data to address the need for timely and adaptive vaccine updates. We propose a framework to guide decisions on vaccine updates, specifically for vaccines that stimulate high-affinity neutralizing antibodies against viral antigens. This framework addresses the challenges of rapidly evolving viruses, where antigenic changes in targets of neutralizing antibody can reduce population-level VE. Using COVID-19 data, we present a real-time VE assessment framework that combines data from SARS-CoV-2 genomic surveillance and VE estimates derived using a test-negative design. We achieve this in three main steps: 1) We explore how genetic distance can serve as an early warning signal for reduced VE, by independently analyzing genomic sequences and epidemiological data. 2) We quantify the relationship between spike gene amino acid distance (amino acid substitutions from COVID-19 case sequences relative to the vaccine formulation(s)) and VE, develop a model capable of predicting VE based on amino acid distance, and validate it using a one-month-out prediction framework; and 3) We generate testable predictions of VE in the absence of individual-level vaccination data, demonstrating that changes in vaccine protection can be inferred from genomic sequences alone.

## Results

**Fig 1:**
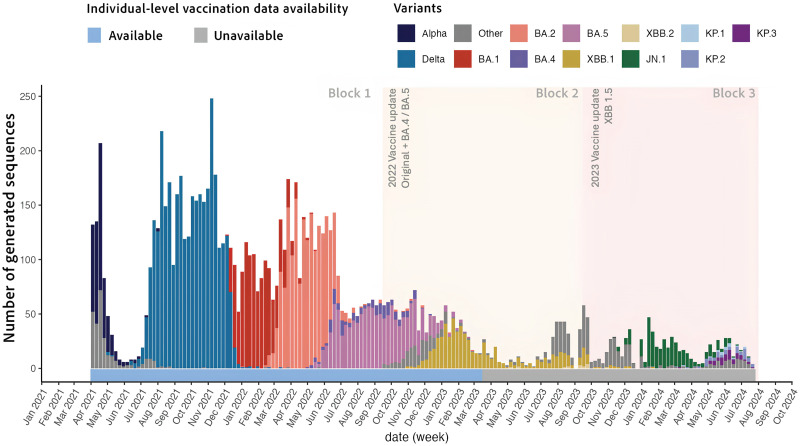
Genomic sequences generated from SARS-CoV-2 cases in Connecticut, US across all vaccine periods (n = 10,156). The number of genomic sequences from SARS-CoV-2 cases is aggregated by week and plotted over the study period, from April 2021 to July 2024. Sequences are colored by different variants of concern (VOCs) prevalent during this period. Background colors represent different vaccine formulation updates: 2020 original monovalent vaccine formulation (white), 2022/2023 bivalent Omicron BA.4/BA.5 formulation (orange), 2023/2024 monovalent XBB 1.5 formulation (pink). The colored bar above x-axis denotes individual-level vaccination data availability: available (light blue) and grey (unavailable).

To investigate how SARS-CoV-2 genetic distance correlates with VE and responds to changes in vaccine formulation over time, we analyzed 10,156 SARS-CoV-2 genomes that we sequenced over 173 weeks through a hospital-based genomic surveillance system (Fig 1). Our longitudinal samples captured most of the variants of concern defined as of October 2024, ranging from Alpha to Omicron KP.3.

The study period also captured three COVID-19 vaccine formulations and was divided into three blocks: 1) the original monovalent mRNA vaccine formulation (April 4, 2021 to September 14, 2022); 2) the bivalent (original formula + BA.4/BA.5) mRNA vaccine formulation (September 15, 2022 to September 14, 2023); and 3) the monovalent XBB.1.5 mRNA vaccine formulation (September 15, 2023 to July 27, 2024).

### Genetic distance as an early warning signal during the original vaccine formulation period

**Fig 2:**
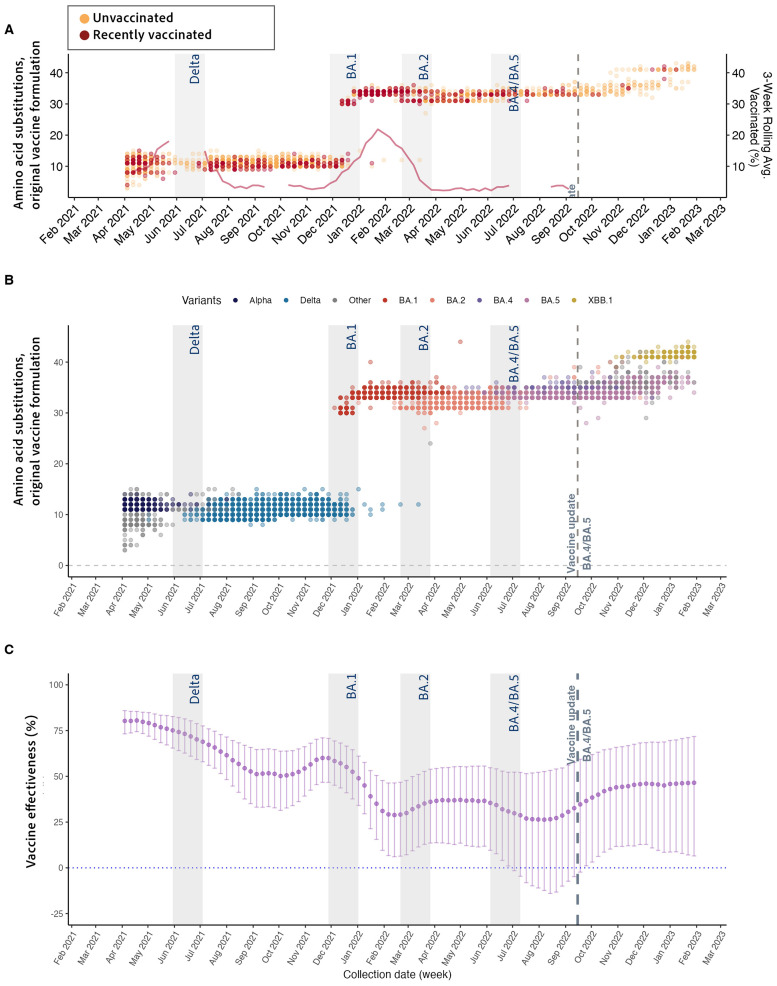
Relationship between spike gene amino acid distance to original vaccine formulation and time-varying vaccine effectiveness for case sequences included in the model calibration, April 2021 to September 2022. Panels (A) and (B) display individual dots, where each dot represents a single sequenced case included in the model calibration. The y-axis indicates the number of amino acid substitutions in the spike gene from the case sequences relative to the original formulation of mRNA COVID-19 vaccines. (A) Comparison of sequences between unvaccinated (yellow) and recently vaccinated individuals (red; 14-90 days since the most recent dose). The red line represents the 3-week rolling average of (%) recently vaccinated individuals among sequenced infections over time; gaps in this line represent intervals with zero observations for this category. (B) Sequences colored by variants of concern assigned to each case sequence (Alpha, black; Delta, blue; Omicron BA.1, red; BA.2, orange; BA.4, purple; BA.5, magenta; XBB.1.5, yellow). Vertical dispersion of dots of the same color reflects within-variant genetic heterogeneity, while the presence of multiple colors in a single week indicates variant co-circulation. (C) Week-level vaccine effectiveness (VE) estimates with 95% credible interval from the time-varying VE model for the period 14-90 days since the most recent dose. Vertical grey boxes denote distinct emergence periods, while the dotted line indicates the introduction of the updated bivalent vaccine formulation.

To evaluate whether SARS-CoV-2 genetic distance can serve as an early warning signal for declining VE at population level, we first investigated both longitudinal genomic and epidemiological data separately. We analyzed 7,722 genomes from cases that we sequenced during the original vaccine formulation period (i.e., block 1) and examined weekly changes across three key aspects (Fig 2).

First, we investigated shifts in the proportion of vaccinated individuals among sequenced infections over time (Fig 2A). We defined vaccine breakthrough cases as individuals who had received at least one vaccine dose 14 or more days prior to a laboratory-confirmed SARS-CoV-2 infection. From mid-January to mid-February 2022, we observed a rapid rise in breakthrough cases among recently vaccinated individuals (i.e., 14–90 days post-last dose) during the Omicron BA.1 period, comprising roughly 20% of all sequences collected weekly (Fig 2A).

Second, we analyzed the weekly average spike gene amino acid distance between the sequenced cases and the vaccine formulation sequence (Fig 2B). During the original vaccine formulation period, we showed that the weekly average genetic distance between case sequences and the vaccine sequence ranged from 10 to 34 amino acid substitutions in the spike gene. During the Delta emergence period, the amino acid distance showed little change compared to earlier variants. However, during the Omicron BA.1 emergence period, we observed that average weekly amino acid distance increased from 10.9 in the first week of December 2021 to 33.3 in the first week of January 2022. The amino acid distance remained relatively stable during the BA.2 and BA.4/BA.5 periods. Within-group variations were also evident both between variants and temporally throughout their respective periods of circulation (S2 Fig). Our analysis of the full spike gene captured a broader range of variations (Fig 2), offering a more comprehensive view than receptor binding domain alone (S3A and S3B Fig). We also tracked temporal changes in nucleotide differences relative to the NCBI reference sequence (S3C Fig). Overall, we demonstrated high correlation between the spike gene amino acid differences used in our main analysis and two alternative measures: restricting the analysis to amino acid substitutions in the receptor binding domain alone (S3B Fig) or extending the analysis to more granular, nucleotide changes within the spike region (S3D Fig).

Third, we examined temporal changes in population-level VE (Fig 2C). Time-varying VE estimates for the period 14–90 days since the most recent dose gradually declined from 80% (95% credible interval (CrI): 74%, 85%) in April 2021 to around 60% spanning the 6-month period before BA.1 emergence. Following the emergence of BA.1, VE dropped sharply from 58% (95% CrI: 47%, 68%) in early December 2021 to 31% (95% CrI: 13%, 46%) by late January 2022. These low VE estimates persisted during the BA.2 and BA.4/BA.5 periods and could not be fully explained by bias from the depletion of susceptibles (S4 Fig). We also showed that after bivalent boosters were introduced in mid-September 2022, which reduced the spike amino acid distance between circulating variants and the vaccine formulation sequence, VE rebounded. In early October 2022, within three weeks of the bivalent booster introduction, mean VE increased by 12 percentage points to 40% (95% CrI: 6.22%, 63.19%), compared to 28% (95% CrI: -9.98%, 54.58%) in late August 2022.

### Quantifying the relationship between amino acid distance and vaccine effectiveness

Based on the observed inverse relationship between the number of amino acid substitutions and VE over time (Fig 2), we hypothesized that population-level amino acid distance in the spike gene can serve as a proxy for vaccine protection. We therefore quantified their direct relationship using our meta-regression model and found that a mean distance of 10 amino acids in the spike gene corresponds to a 15.4% (95% CrI: -2.00%, 34.6%) decrease in predicted VE.

**Fig 3:**
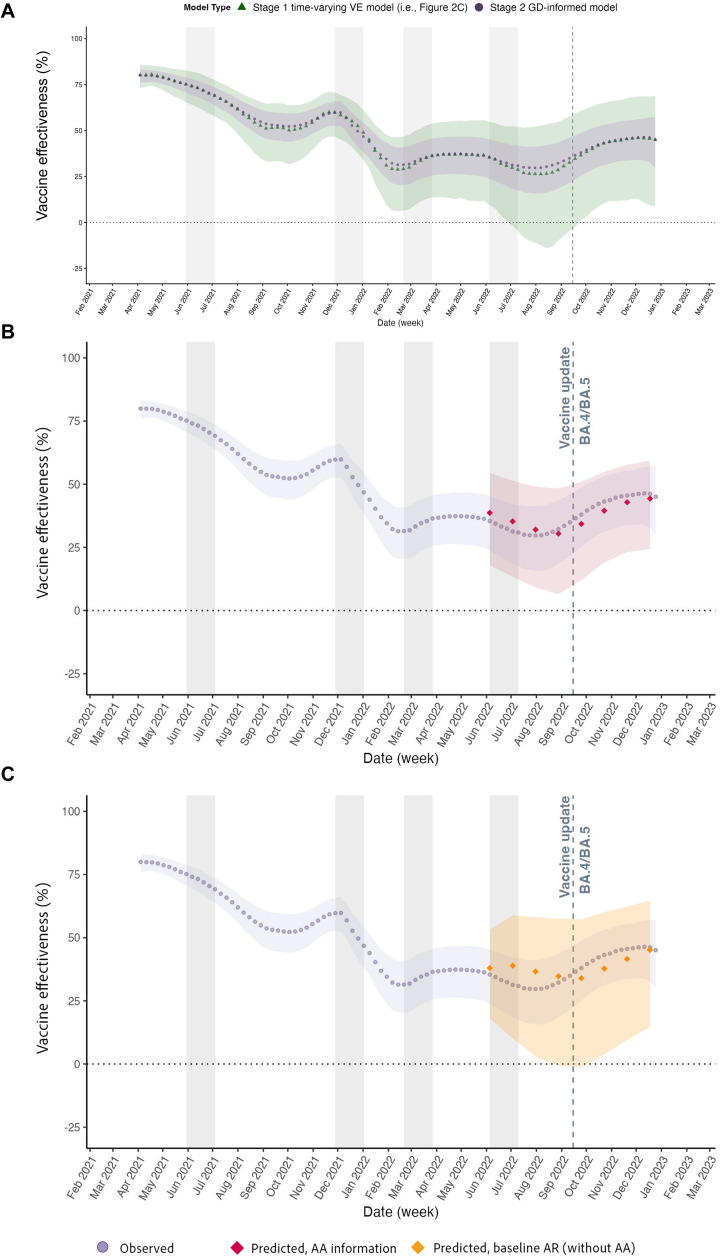
Comparison of model stages and validation of one-month-ahead vaccine effectiveness (VE) predictions. **(A)** Comparison of first-stage time-varying VE estimates and corresponding 95% credible intervals (CrI) (i.e., Fig 2C) in green and “observed” VE estimates from the amino-acid-distance meta-regression model in purple. Vertical grey boxes denote variant emergence periods, and the dotted line indicates the bivalent vaccine introduction. (B - C) Validation of the observed VE (purple ribbons carried over from panel A) to **(B)** one-month-ahead predicted VE from the amino-acid-distance meta-regression model (red diamonds), which uses week-level amino acid distance as input, and (**C**) the baseline meta-regression model (orange diamonds), which does not incorporate amino acid distance information. Colored ribbons represent 95% CrI for observed (purple) and predicted (red and orange) values.

To validate the model’s ability to predict VE using weekly amino acid distance data, we generated one-month-out predictions and compared them to observed VE values (Fig 3). We found that the model closely followed the observed VE trends across all prediction months (mean absolute error (MAE) = 3.33) and successfully captured changes surrounding the bivalent vaccine update. All VE values during the eight-month prediction window—four months before and after the vaccine update—fell within the 95% prediction intervals (Fig 3B). We also showed that this model outperformed a reference model that had the same autoregressive structure but excluded amino acid distance information (Fig 3C); the baseline AR model yielded a higher MAE (MAE = 5.86) and a higher continuous ranked probability score (CRPS) throughout most of prediction months (S5 Fig). Additionally, the baseline model produced markedly wider prediction intervals, reflecting increased uncertainty in its prediction. These findings highlight the added value of incorporating genomic information in enhancing the model's accuracy in predicting future VE compared to a model relying solely on historical VE data.

### Estimating changes in vaccine effectiveness for the XBB.1.5 vaccine formulation: simulating real-world scenarios without vaccination data

After calibrating and validating our model (Fig 3), we then sought to translate these findings into a real-world framework to inform changes in predicted vaccine protection as new variants emerged. To evaluate how population-level amino acid distance from the sequence of different COVID-19 vaccine formulations changed across multiple vaccine updates, we extended our model across the different formulation periods. Throughout vaccine update blocks, we utilized only measured weekly amino acid distance as the input to predict the percent reduction in VE; we present VE estimates based on a 3-week rolling average of amino acid distance to enhance visualization.

**Fig 4:**
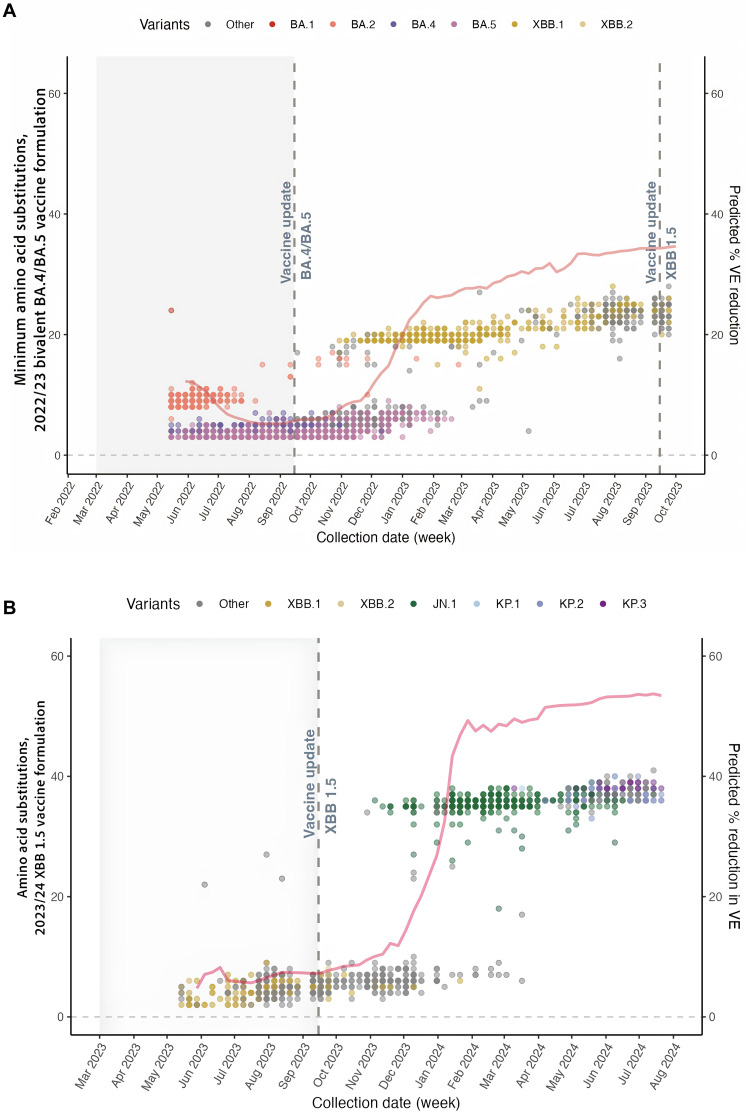
Amino acid differences in spike gene between case strains and the updated formulation of mRNA COVID-19 vaccines. **(A)** Weekly minimum amino acid substitutions in the spike gene between case strains and either of the two strains included in the bivalent mRNA COVID-19 vaccine formulation (May 2022 – September 2023). **(B)** Amino acid differences relative to the XBB.1.5. formulation of mRNA COVID-19 vaccines (May 2023 – July 2024). In both panels, each dot represents a single sequenced case and sequences are colored by different variants of concern prevalent during this period. The grey dotted line represents the introduction of the vaccine update. The grey shaded area represents sequences that were collected prior to updated vaccine availability. The red line represents the weekly mean predicted percent reduction in VE based on the 3-week rolling average of amino acid distance from the vaccine strain.

During the second vaccine (block 2), the increase in minimum amino acid distance to either formulation was more nuanced than in block 1. Block 2 was characterized by the prolonged co-existence of BA.5 and XBB strains, which led to a gradual rather than step-wise increase in population-level mean amino acid distance (Fig 4A). For instance, with the emergence of XBB strains, the mean distance rose from 12.2 in late-December 2022 to 18.2 in late-January 2023 and 23.7 by mid-September when the 2023/24 XBB vaccine (i.e., third vaccine) became available, marking the first annual COVID-19 vaccine update. This increase in amino acid distance predicted a 34.3% (95% CrI: -2.4%, 70.4%) reduction in VE against the second vaccine (2022/23 vaccine strains) by mid-September 2023.

For the third vaccine update (block 3), we observed a more than two-fold increase in mean amino acid distance, mirroring the pattern seen during the original vaccine formulation period (Fig 4B). Specifically, the mean amino acid distance rose from 12.3 in mid-December 2023 to 30.2 in mid-January 2024, corresponding to the transition from XBB to JN.1 dominance. Based on this increase in amino acid distance, we predicted a 43.4% (95% CrI: -5.7%, 90.1%) reduction in VE by mid-January 2024. Our findings also suggest a further 9.8% decline in VE against circulating variants by June 2024, coinciding with the emergence of KP lineages, reaching an estimated 53.2% (95% CrI: 7.0%, 110.4%) reduction compared to VE against the XBB.1.5 vaccine strain.

To assess the generalizability of our findings, we evaluated temporal changes in amino acid distance across the Northeast United States using all publicly available sequences from the NCBI database. Although the precise timing of the abrupt increase in mean amino acid distance varied by state, the overall evolutionary trajectory of circulating strains relative to the vaccine formulation closely mirrored our observations in Connecticut (S6 Fig). This approach demonstrates how the model can support real-time surveillance by quantifying expected declines in vaccine protection as new variants emerge.

## Discussion

Our analysis estimated time-varying COVID-19 VE at the population level and quantified its direct relationship with SARS-CoV-2 genomic data from community cases. The time-varying VE framework that we introduce is useful by itself for identifying and predicting temporal changes in VE. However, complementing the framework with genomic information provides additional precision for generating one-month-ahead predictions of VE. We found that virus genomic data, in the form of amino acid distance from the vaccine formulation sequence, can serve as an early warning to detect shifts in predicted VE and provides insights into the underlying causes of these changes. Combining time-varying VE estimates with the association between VE and amino acid distance allows us to quantify how the emergence of new variants is expected to affect VE.

We demonstrated a clear association between increasing amino acid distance in the SARS-CoV-2 spike gene from the vaccine formulation and declining population-level VE. This aligns with a growing body of research highlighting the critical role of spike mutations on antibody neutralization and immune escape [22–24]. While many prior works on immune escape have focused on variant-specific analyses [25], *in vitro* characterization of specific mutations [26], or mapping of viral evolution across variants using antigenic cartography [27], our approach offers a complementary population-level perspective. We provide a variant-agnostic and practical metric for real-time monitoring of potential reductions in VE. This contrasts with traditional VE studies that often rely on retrospective analyses of observational data.

For SARS-CoV-2, genetic distance to the vaccine sequence has previously been explored as a correlate of vaccine efficacy based on clinical trial data [28,29] and through predictive models using publicly available data [30]. Previous models focused on forecasting and characterizing specific mutations of concern [30]; our study expands the application by directly estimating VE using genomic surveillance data. A key strength of our study lies in the comprehensive surveillance data, which encompasses four years of SARS-CoV-2 genomic surveillance from a single hospital system, uniquely linked to detailed clinical metadata. This integration of epidemiological and genomic data—rather than relying on public databases—allows us to quantify the relationship between VE and temporal changes in amino acid distance, advancing our understanding of vaccine protection in an evolving antigenic landscape. Furthermore, our Bayesian approach to estimating time-varying VE with autoregressive terms enables us to provide robust and informative estimates even for weeks with limited sequence availability.

Vaccine formulation updates are sometimes necessary to provide sufficient protection against emerging immune escape variants [31,32]. In the case of SARS-CoV-2, the emergence of antigenically distinct lineages has been characterized by the accumulation of a large number of mutations, which thus far has been difficult to predict [2]. COVID-19 vaccine formulation updates have generally been implemented annually since 2023. However, the degree of antigenic divergence from the existing vaccine formulation varied among the variants that prompted these updates. For instance, the emergence of Omicron lineages, characterized by over 30 amino acid substitutions in the spike protein alone compared to ancestral lineages, prompted the first vaccine update [33,34]. Our analysis showed that an increase in amino acid distance of approximately 30 substitutions following the emergence of Omicron BA.1 corresponded to an estimated 40% decrease in VE. The emergence of JN.1 variants in November-December 2023 was characterized by a similarly large increase in amino acid distance relative to the XBB.1.5 (2023/24 vaccine update) formulation, which we predict should have resulted in a similar ~40% decrease in VE. However, the antigenic divergence of XBB.1.5 from the preceding BA.4/BA.5 vaccine (2022/23 update) resulted in comparatively smaller predicted VE reduction, estimated at ~20% upon emergence in late December 2022 and ~35% when the updated vaccine was implemented (September 2023). Our framework could help identify when emerging variants are associated with large (vs small) declines in VE, better guiding when COVID-19 vaccine formulation updates are needed, as opposed to the current practice of updating the vaccine annually. This could enable preparedness efforts before clinical data fully accumulate, allowing for a more timely and dynamic response system in the future. The threshold amino acid distance dictating vaccine updates can be adjusted based on desired vaccine protection targets, but the key insight is that waiting for full laboratory and clinical confirmation may not be necessary to initiate a response. Instead, amino acid distance can serve as an effective early indicator, with additional data validating and refining decisions.

Limitations to our study include the lack of disease severity information among outpatients; therefore, our VE estimates are not outcome specific (e.g., hospitalization, death, etc.) within this population. While outcome-specific VE would provide further insights, our focus on outpatient data aligns more closely with the population we aimed to study, namely people seeking healthcare due to COVID-19-related symptoms in the community setting. Inpatient data were excluded due to the potential for bias introduced by routine testing protocols prior to procedures (e.g., surgeries). Similarly, we identified individuals with high frequency of testing (more than 50 times between April 2021 and March 2023) as likely undergoing routine testing. Although we made efforts to flag and account for their potential influence in our models, some residual bias may remain. Such individuals may be more likely to test negative and either more likely to be vaccinated (if they represent very “COVID-aware” individuals) or less likely to be vaccinated (if routine testing was required by their employer as a condition of being unvaccinated). Furthermore, our VE estimates are not vaccine specific. We used the mRNA vaccine sequences for the Pfizer BNT162b2 vaccine, which were similar for the Moderna vaccine, to define amino acid distance. While these vaccines may not fully represent all COVID-19 vaccines used in our study population, they account for over 97% of doses administered in the US [35–37]. Additionally, we did not account for potential bias related to the depletion of susceptible people, which can occur when natural immunity from infection is acquired faster in the unvaccinated group than in the vaccinated group, potentially underestimating VE and leading to incorrect conclusions about time-varying effectiveness [38,39]. We mitigated this potential bias by focusing our primary analysis on recently vaccinated individuals (14–90 days), thereby capturing VE estimates before differential depletion could significantly skew the results [40]. Furthermore, the high reinfection rates of emerging variants provide a natural buffer against sustained depletion bias; as immune escape variants allow individuals with previous infection-induced immunity to re-enter the susceptible pool, this “refilling” effect reduces statistical artifacts associated with spurious waning [40], as demonstrated by our simulation-based sensitivity analysis. Finally, we implemented a two-stage approximation to a full hierarchical Bayesian model. While this remains an approximation, we addressed first-stage uncertainty through a meta-regression framework with autoregressive random effects. Although this approach likely resulted in wider credible intervals, it prevented the model from overestimating precision and minimized the risk of Type I errors. While specific VE estimates may vary depending on the population and setting, our approach provides a framework that is adaptable to other contexts, provided genomic surveillance data are available and can be linked to patient information for cases and controls.

Uncertainties remain in the seasonality of SARS-CoV-2 [41,42] and the optimal frequency of COVID-19 vaccine updates. Our study provides a framework for the early identification of emerging variants associated with declines in vaccine protection, which could allow for more adaptive decision-making on COVID-19 vaccine updates. Currently, committees like the ACIP play a pivotal role in making policy recommendations based on comprehensive review of clinical, epidemiological, safety, and economic data [12]. Our framework supplements current work as we demonstrate how genomic data can be integrated with more traditional epidemiological data sources to address the need for timely, reliable evidence to guide vaccine updates. It also provides reassurance during periods when the current vaccine is expected to remain effective, strengthening public confidence in VE messaging. Ultimately, vaccine protection is shaped by a complex interplay of factors, including vaccine properties, evolutionary dynamics of the virus, biological factors influencing host immunity, and selective pressure within a population [41]. This highlights the need for continuous monitoring and robust data. Genomic surveillance is integral to this process, enabling more informed decision-making and effective public health strategies.

## Materials and methods

### Ethics statement

The study protocols for pathogen genomic sequencing of remnant diagnostic samples (IRB Protocol ID: 2000033281) and impact of SARS-CoV-2 genetic variants on immune escape and vaccine breakthroughs (IRB Protocol ID: 2000031374) was reviewed and approved by the Yale University institutional review board.

### Data sources and genetic sequences

We used whole genome sequencing (WGS) data collected from the Yale New Haven Hospital (YNHH) system in Connecticut (CT), US between the week of 4^th^ April 2021 to the week of 21^st^ July 2024 through the Yale SARS-CoV-2 Genomic Surveillance Initiative. Sample collection, RT-qPCR, and sequencing protocols have been described in detail elsewhere [43,44]. Metadata and vaccination records were obtained from the YNHH system and Center for Outcomes Research and Evaluation and matched to WGS data using unique sample identifiers. We used standardized ZIP-code-level CT income data as a potential covariate, with raw data obtained from the US Census Bureau [45].

We highlighted emergence periods for better visualization and understanding of variant timelines. For uniformity, we applied the same emergence period definitions previously established in Chen et al.[44] In short, these periods represent short intervals during which two variants co-circulated, defined as the time from when a variant accounted for 5% of Global Initiative on Sharing All Influenza Data (GISAID)-reported cases in CT to when it reached its maximum frequency in the population [44].

### Epidemiological data

Our study focused on CT residents to ensure consistency in surveillance and vaccination policies. Cases were defined as individuals who tested positive for SARS-CoV-2 infection via PCR and who had samples sequenced at the WGS level. To maintain a consistent definition of VE, only samples taken from outpatient settings were included. We excluded samples for whom WGS information was not available or did not meet the required genome sequence coverage (i.e., genome coverage >95%). Other exclusion criteria included duplicate samples from an individual (to exclude chronic infections) and samples from non-CT residents. Given the vaccine rollout and eligibility timelines differed across age groups, we only included adult patients (18 + years) at the time of sample collection. Cases were aggregated at the week level to reduce sparsity, and subsequent analyses were conducted at the week level.

To assess VE over the first 103 weeks, corresponding to the first two years of the data collection (April 4, 2021 to March 19, 2023), we obtained individual-level metadata from test-negative controls. Controls were selected from samples taken from individuals in outpatient settings who met all criteria for the YNHH system testing initiative, but who tested negative for SARS-CoV-2 infection via PCR. If an individual had both negative and positive test results, we only included the positive test as a case. Any subsequent negative test from the same individual within two weeks of a positive test was excluded to avoid potential misclassification.

A patient’s vaccination status was determined at the time of sample collection, and time since last vaccination was calculated as the difference between the sample collection date and date of last vaccine receipt. Primary analyses were conducted based on time since last vaccination to account for waning immunity, and uniform criteria were applied regardless of the vaccine manufacturer or number of doses received. Time since last vaccination was categorized as: < 14 days, 14–90 days, 91–180 days, ≥181days. The total number of doses received more than 14 days prior to sample collection was also counted for each individual and included as a covariate in our model. For visualization, we plotted 3-week rolling averages to show the proportion of recently vaccinated individuals among both the cases with collected sequences and test-negative controls over time. This allows us to observe trends in vaccine coverage in both groups.

To ensure that our controls were representative of the population, vaccine coverage among controls was compared against available population vaccine coverage data in CT (S1 Fig). We obtained data on the CT vaccination trends from the CDC [46].

### Vaccine updates and corresponding sequences

Comirnaty (Pfizer-BioNTech) or Spikevax (Moderna Tx.) were the most widely available and used COVID-19 vaccines in the US. Updates to these two mRNA-based vaccines mostly followed a similar timeline throughout the study period with three updates as of October 2024 [47].

Vaccine blocks were defined based on FDA approval dates, though the exact cut-off dates were selected arbitrarily for analysis purposes. The FDA authorized the use of the bivalent (original formula + BA.4 / BA.5) vaccine as a booster dose on August 31, 2022 [48]; we assigned September 15, 2022 as the start of bivalent vaccine availability, regardless of the vaccine manufacturer [49]. The monovalent XBB.1.5 formulation was approved on September 11, 2023 [50]; we assigned September 15, 2023 as the beginning of the third block.

### Genetic distance model

We defined genetic distance as the number of amino acid substitutions between the case and mRNA vaccine formulation spike gene sequences. We aligned sequences using nextclade [51], extract the spike gene region from aligned WGS, we used bedtools [52] to trim the specified genomic coordinates at the nucleotide level. We then translated the nucleotide sequences to amino acid. We measured genetic distance using Hamming distance, which calculates the number of mismatched positions between aligned sequences [53]. Hamming distance was selected as it has been shown to correlate well with antigenic distances and has been effectively used in influenza vaccine research [53–55]. For nucleotide-level comparisons, we calculated the distance between each case sequence and the NCBI reference genome (MN908947), the basis for the original vaccine formulation.

For each vaccine block, we compared case sequences to the corresponding vaccine sequence available during that time period. For example, sequences from block 1 were compared against the original mRNA vaccine sequence, while sequences from block 3 were compared against the 2023/2024 XBB.1.5 formulation. For block 2, we used the minimum amino acid differences between the case strain and either of the two strains in the bivalent vaccine.

### Time-varying vaccine effectiveness (VE) model

We assessed time-varying VE using a hierarchical Bayesian time-series method, building on the widely used test-negative design that compares the prevalence of vaccination among SARS-CoV-2 cases and test-negative controls. The binary outcome variable for an individual *i* at time *t* ($Y_{it})\;$is equal to one if the individual is a case and zero if they are a control. We used logistic regression to model the probability of being a case or control for each individual and time point ($p_{it}$) as a function of vaccination status and other covariates. Additionally, we extended the conventional test-negative design approach by incorporating correlated random effects to allow the association between vaccination status and case/control probability to smoothly change across time. Specifically, the model is given as:


$$\begin{array}{c} Y_{it}|p_{it}\;\sim \text{ Bernoulli}\left(p_{it}\right),\text{ logit}\left(p_{it}\right)=\;\beta_{\text{0}t}+\;\sum_{j=\text{1}}^{c}\beta_{jt}{\text{Z}}_{ijt}+\overset{\text{T}}{\underset{i}{𝐱}}\eta \end{array}$$ (1)

where $\beta_{\text{0}t}$ is the time-varying intercept parameter that accounts for changes in baseline risk of SARS-CoV-2 infection for each week *t*; $Z_{ijt}$ is an indicator variable for the vaccination ca*t*egory (0 (reference): unvaccinated, 1: < 14 days, 2: 14 – 90 days, 3: 91 days – 6 months, 4: > 6 months) of individual *i* in week *t*; $\beta_{jt}$ is the time-varying regression parameter that describes the association between vaccination ca*t*egory *j* and the probability of being a case; and ${𝐱}_{i}$ is a vector of covariates for individual *i* for which the corresponding effect estimates do not change across time. The vector of other covariates includes age group, standardized income at the zip-code level, gender, number of vaccine doses received before sample collection, and a “routine flag” (binary variable) to indicate individuals who are thought to be testing routinely (defined as 1 for individuals with more than 50 testing records over the 103 weeks included in our dataset and 0 otherwise).

The time-varying parameters, $\beta_{0t}$ and$\;\beta_{jt}$, were modeled as a function of a “global” fixed effect parameter and independent (across parameter) autoregressive correlated random effects, such that:


$$\begin{array}{c} \beta_{jt}=\left(\beta_{j}+\theta_{jt}\right);\;\theta_{jt}|\;\theta_{jt-\text{1}},\;\rho_{j},\overset{\text{2}}{\underset{j}{\tau }}\;\sim \text{ N}\left(\rho_{j}\theta_{j,t-\text{1}},\;\overset{\text{2}}{\underset{j}{\tau }}\right),j=\text{0},\ldots ,c. \end{array}$$

The autoregressive structure specified for the $\theta_{jt}$ parameters allows both intercept and coefficients to evolve dynamically while borrowing strength across nearby time points. We allow the data to determine the strength of temporal correlation by estimating the $\rho_{j}$ parameters. To complete the model structure, we assigned prior distributions to the remaining model parameters. Specifically, we assigned independent log-gamma(3, 2) priors to the precision parameters $\left(\frac{\text{1}}{\overset{\text{2}}{\underset{j}{\tau }}}\right)$ of the random effects. For the fixed effects, including η and $\beta_{j}$, we used weakly informative Normal(0, ${\text{1}\text{0}}^{\text{3}}$) priors. Additionally, we selected independent Uniform(-1, 1) distributions for the autoregressive parameters ($\rho_{j}$).

We used R-INLA (integrated nested Laplace approximations) (www.r-inla.org) [56,57] to fit the model. R-INLA is a computationally efficient approximation to a widely used Markov chain Monte Carlo (MCMC) approach, and provides marginal posterior inference for the included model parameters [58]. We generated 10,000 samples from an approximated marginal posterior distribution for each parameter in parallel using pbmcapply and parallel R packages. Along with posterior means of $\beta_{jt}$, the quantile based, equal tailed 95% credible intervals (CrI) of $\beta_{jt}$ were constructed for each vaccination category. For better visualization, both the posterior mean and CrI for 1- exp${(\beta }_{jt})$ were also calculated and plotted, representing VE (%) at each week.

### Quantifying the relationship between amino acid distance and vaccine effectiveness

We used a random effects meta-regression model fitted in the Bayesian setting to quantify the relationship between amino acid distance and VE over time, focusing on the 14–90 days post-vaccination category (*j = 2*) to represent short-term vaccine-induced immunity. We modeled the outcome variable, the observed log OR of infection at time *t* (${\hat{\beta }}_{\text{2}t}$) from the time-varying VE model as following a normal distribution centered around the latent true log OR ($\beta_{\text{2}t}$), with variance defined by the squared posterior standard deviations (${{\hat{\sigma }}_{t}}^{\text{2}}$). Then, the true but unobserved log OR is modeled as a function of amino acid distance and random effects. Specifically, the full model is given as:


$$\begin{array}{c} {\hat{\beta }}_{\text{2}t}|\beta_{\text{2}t}\;\sim \text{ N }\left(\beta_{\text{2}t},\;{{\hat{\sigma }}_{t}}^{\text{2}}\right);\;\beta_{\text{2}t}=\;\gamma_{\text{0}}+\gamma_{\text{1}}{AA}_{t}+\alpha_{t}+\varepsilon_{t} \end{array}$$

where $\gamma_{\text{1}}$ is the key regression parameter that quantifies the association between amino acid distance and the relative risk of infection; $\gamma_{\text{0}}$ is the baseline intercept that accounts for variations in baseline risk across all time points; $\alpha_{t}$ captures time-varying random effects with an autoregressive structure similar to the first-stage model; and $\varepsilon_{t}$ is an independent error term that follows a zero-mean normal distribution. To complete the model structure, we assigned weakly informative priors to the remaining parameters. Specifically, we assigned Normal(0, ${\text{1}\text{0}}^{\text{3}}$) priors for the fixed effects, including $\gamma_{\text{0}}$ and $\gamma_{\text{1}}$. For the random effects ($\alpha_{t}$), we assigned a Uniform(-1, 1) prior to the autoregressive parameter ($\rho_{A}$), consistent with our first-stage model, and a Gamma(0.01, 0.01) prior to the precision parameter governing the variability.

We used MCMC methods to fit the model and sampled from the joint posterior distribution of all model parameters using the rjags package [59] in R. We initialized four MCMC chains with an adaptive phase of 500,000 iterations, followed by 100,000 iterations after convergence. Convergence was assessed via Geweke diagnostics, which yielded Z-scores of -0.648 for $\gamma_{0}$ and 0.675 for $\gamma_{1}$, indicating no obvious signs of non-convergence. Posterior inference was based on posterior means and a 95% CrI for key parameters including $\gamma_{1}$.

#### Model validation and prediction.

We assessed the predictive performance of the random effects meta-regression model using a one-month-out validation framework. This approach was designed to simulate real-world scenarios where genomic surveillance data are available, but individual-level patient metadata are limited. In such contexts, amino acid distance data serve as the primary input for predicting VE, which is derived from estimates of $\beta_{\text{2}t}$ from the meta-regression model.

We generated predictions over an eight-month time period from June 2022 to January 2023, spanning four months before and after the bivalent vaccine update. Specifically, for each held-out month *k*, we excluded the observed ${\hat{\beta }}_{2t}$ values from the model and used only amino acid distance data for that month as input. The observed ${\hat{\beta }}_{2t}$ values from months 1 to *k*-1 were retained in the model to inform the posterior distribution of the time-varying parameters. The model was used to predict $\beta_{2t}$ for month *k*, given the amino acid distance data for that month. For example, to predict the $\beta_{2t}$ for June 2022, the model was given ${\hat{\beta }}_{2t}$ data up to May 2022 and was asked to predict the $\beta_{2t}$ for June 2022 based on the amino acid distance data for June 2022. This process was repeated for each month in the validation period.

We fit the meta-regression model described in the previous section separately for each leave-out month using MCMC methods. We implemented the model using four MCMC chains with 50,000 iterations followed by 10,000 iterations after convergence. The model’s predicted $\beta_{2t}$ values were compared to the observed ${\hat{\beta }}_{2t}$ to assess its predictive performance. The MAE was used as the primary metric to quantify prediction error. A baseline autoregressive model, using a similar one-month-out validation framework and MCMC settings, was also evaluated; this model did not incorporate amino acid distance information, instead predicting VE based only on the autoregressive terms.

To compare the predictive performance of these two models, we calculated CRPS. Unlike the MAE, which compares point predictions to observed values, the CRPS is a generalized MAE that evaluates the entire predictive distribution [60,61]. We computed CRPS for each one-month-out prediction for both models to provide a more comprehensive assessment of their probabilistic forecasting capabilities.

#### Simulation-based sensitivity analysis.

To address concerns over the potential magnitude and timescale of bias in estimates of the time-varying VE due to the differential depletion of susceptible unvaccinated versus vaccinated individuals [38,39], we developed a simple compartmental SIRS model simulation using parameters that resemble SARS-CoV-2-like characteristics (S1 Table). We compared two scenarios:

1. Model 1 (SIRS / refilling pool): Recovered individuals from both vaccinated and unvaccinated groups re-enter susceptible pool (waning immunity: 90 days). This model reflects the high reinfection rates characteristic of the Omicron era.
2. Model 2 (SIR/ Sustained depletion): This model assumes that recovered individuals remain immune.

The model diagrams and equations are provided in the S1 File.

Our results demonstrate that across a range of transmission intensities, Model 1 (which is more representative of the short duration of immunity against reinfection with SARS-CoV-2) consistently yields VE estimates that track close to the true VE. The estimated VE initially dips below the true VE (~75%) as differential depletion begins but recovers toward the true VE (~90%) as the susceptible pools are progressively replenished. This non-monotonic behavior demonstrates that the refilling mechanism effectively attenuates the bias over the course of the epidemic. In contrast, Model 2 (in which we assume no waning of immunity) leads to a progressive underestimation of VE as the susceptible denominator is artificially exhausted. In the absence of waning immunity, the bias is sustained and monotonically increasing; the estimated VE progressively diverges from the true VE as unvaccinated susceptible individuals are preferentially depleted.

## Supporting information

S1 Fig Vaccine coverage trends in Connecticut (April 2021 – May 2023). Weekly vaccine coverage data for Connecticut, USA, based on publicly available CDC data. Colors indicate different administered dose categories: dose 1, primary series completion, and additional doses. Coverage is presented as the percentage of the population receiving each dose type over time. Coverage among cases (blue line) is presented as 3-week rolling average.
(TIF)

S2 Fig Temporal dynamics of within-variant heterogeneity. The panels display the 30-day rolling standard deviation of amino acid substitutions in the spike gene, faceted by variants of concern.
(JPG)

S3 Fig Amino acid differences in the receptor binding domain relative to the original mRNA COVID-19 vaccine strain, April 2021 to September 2022. (A) Weekly aggregated amino acid differences in the receptor binding domain region between the case strain and the original formulation of mRNA COVID-19 vaccines; each dot represents a single sequenced case. (B) Correlation between amino acid differences in the spike gene and receptor binding domain. (C) Weekly aggregated nucleotide differences in the spike gene between the case strain and reference SARS-CoV-2 sequence (NCBI Accession: MN908947). (D) Correlation between amino acid differences in the spike gene and nucleotide differences in the spike gene. Sequences are colored by the predominant variants of concern prevalent during this period (Alpha, black; Delta, blue; Omicron BA.1, red; BA.2, orange; BA.4, purple; BA.5, magenta). Spearman correlation coefficients are annotated in panels (B) and (D).
(JPG)

S4 FigSimulation-based sensitivity analysis results.Figure displays the SIRS-model simulation results for model 1 (blue), which assumes recovered individuals from both vaccinated and unvaccinated groups re-enter susceptible pool (waning immunity: 90 days), and model 2 (maroon), which shows no waning from the recovered state. Results are shown for three different effective reproductive number ($R_{eff}$). The horizontal dotted line represents the assumed “true” vaccine effectiveness parameter.(TIF)

S5 Fig Continuous ranked probability score (CRPS) model comparison. Figure displays the CRPS values for the amino acid (AA) distance informed model (red line) and the baseline autoregressive (AR) model (orange) over the eight-month prediction and validation period. Lower CRPS values indicate better performance.
(TIFF)

S6 Fig Amino acid substitutions in spike gene between case strains in the Northeast US (n = 49,266) and the XBB. 1.5 formulation of mRNA COVID-19 vaccines. Sequences are aggregated by week from Jun 15, 2023, to August 30, 2024. Each dot represents a single sequenced case. The grey dotted line represents the introduction of the XBB.1.5. vaccine update. The grey shaded area represents sequences that were collected prior to XBB.1.5 vaccine availability. (A) All northeast sequences combined and colored by Nextclade lineage assignment. (B) Comparison of mean amino acid substitutions over time between Northeast sequences (red) versus Connecticut (blue) sequences. (C) State-level comparisons, where sequences from each of the Northeast states are plotted against Connecticut (blue) sequences to highlight local variations in the timing and magnitude of amino acid substitutions.
(JPG)

S1 Table Fixed model parameter values. (DOCX)

S1 File Supplementary Methods. (DOCX)

S1 Data EPI_SET ID for the sequences used in the study. (CSV)
