# Supplementary figures and images for: Utilizing virus genomic surveillance to predict vaccine effectiveness
Source: PLoS Comput Biol. 2026 May 26;22(5):e1014329. doi: 10.1371/journal.pcbi.1014329 (PMC13258143; doi:10.1371/journal.pcbi.1014329)

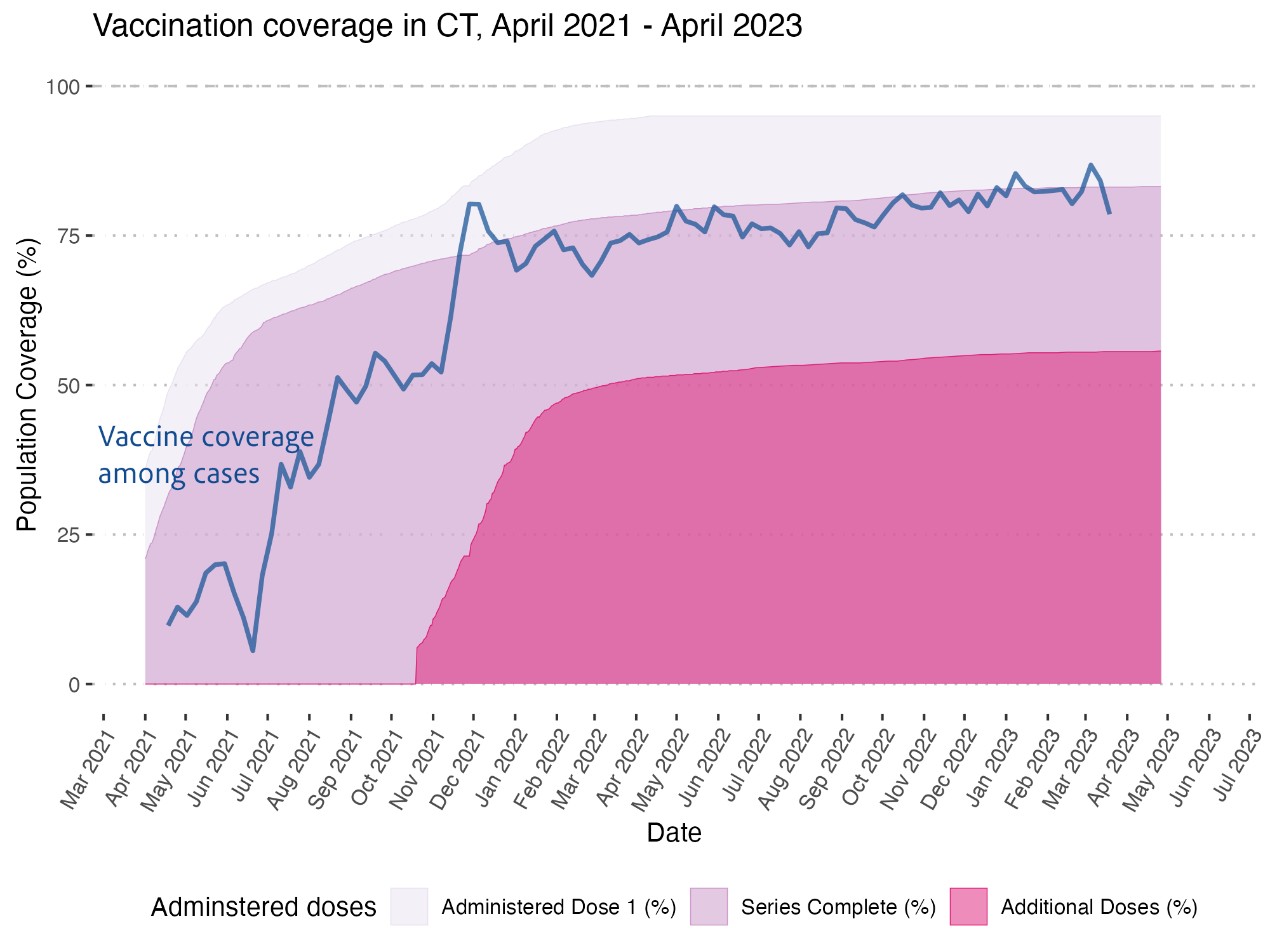

Supplement: S1 Fig — Weekly vaccine coverage data for Connecticut, USA, based on publicly available CDC data. Colors indicate different administered dose categories: dose 1, primary series completion, and additional doses. Coverage is presented as the percentage of the population receiving each dose type over time. Coverage among cases (blue line) is presented as 3-week rolling average. (TIF) [file pcbi.1014329.s001.tif]

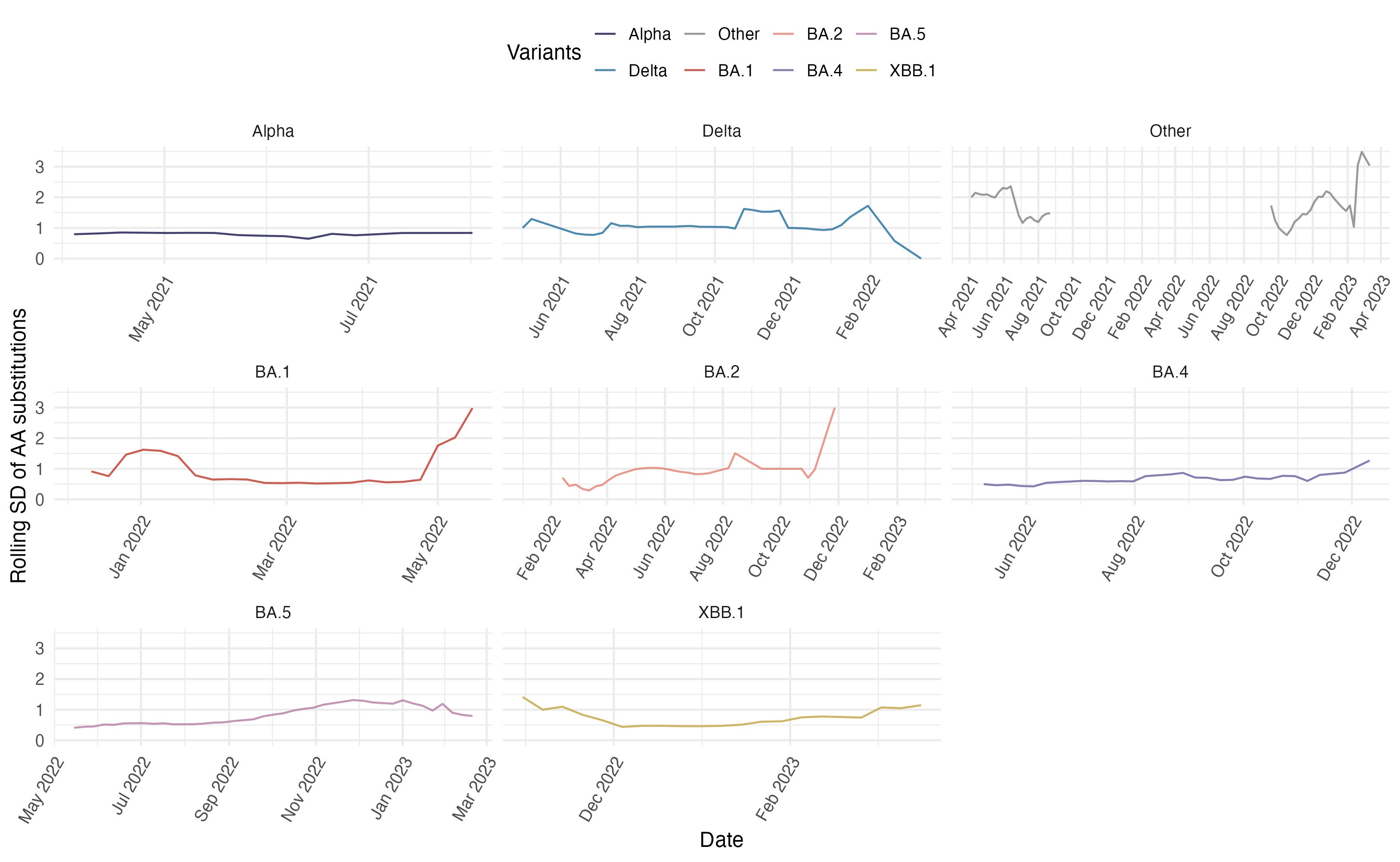

Supplement: S2 Fig — The panels display the 30-day rolling standard deviation of amino acid substitutions in the spike gene, faceted by variants of concern. (JPG) [file pcbi.1014329.s002.jpg]

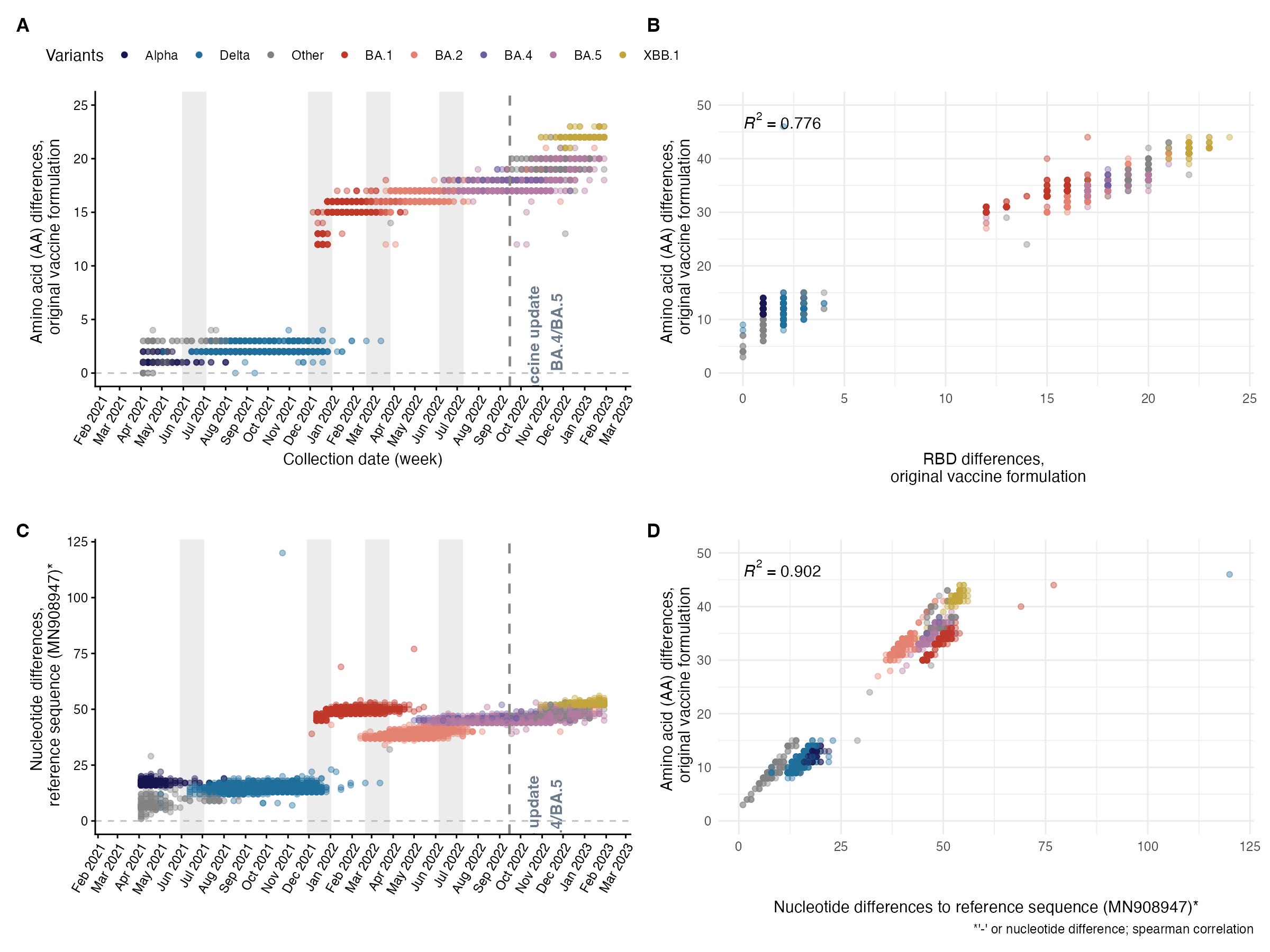

Supplement: S3 Fig — (A) Weekly aggregated amino acid differences in the receptor binding domain region between the case strain and the original formulation of mRNA COVID-19 vaccines; each dot represents a single sequenced case. (B) Correlation between amino acid differences in the spike gene and receptor binding domain. (C) Weekly aggregated nucleotide differences in the spike gene between the case strain and reference SARS-CoV-2 sequence (NCBI Accession: MN908947). (D) Correlation between amino acid differences in the spike gene and nucleotide differences in the spike gene. Sequences are colored by the predominant variants of concern prevalent during this period (Alpha, black; Delta, blue; Omicron BA.1, red; BA.2, orange; BA.4, purple; BA.5, magenta). Spearman correlation coefficients are annotated in panels (B) and (D). (JPG) [file pcbi.1014329.s003.jpg]

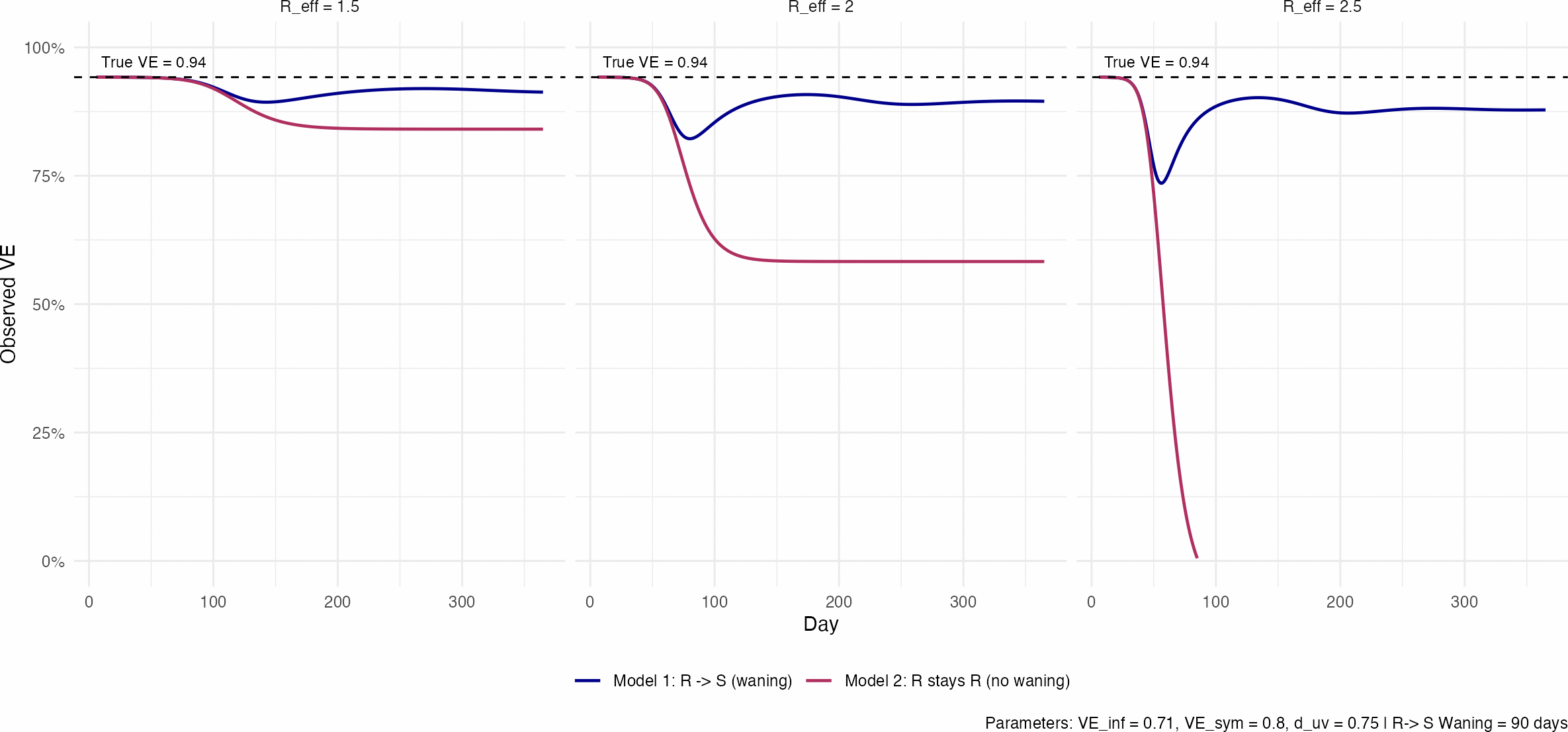

Supplement: S4 Fig — Figure displays the SIRS-model simulation results for model 1 (blue), which assumes recovered individuals from both vaccinated and unvaccinated groups re-enter susceptible pool (waning immunity: 90 days), and model 2 (maroon), which shows no waning from the recovered state. Results are shown for three different effective reproductive number (Reff). The horizontal dotted line represents the assumed “true” vaccine effectiveness parameter. (TIF) [file pcbi.1014329.s004.tif]

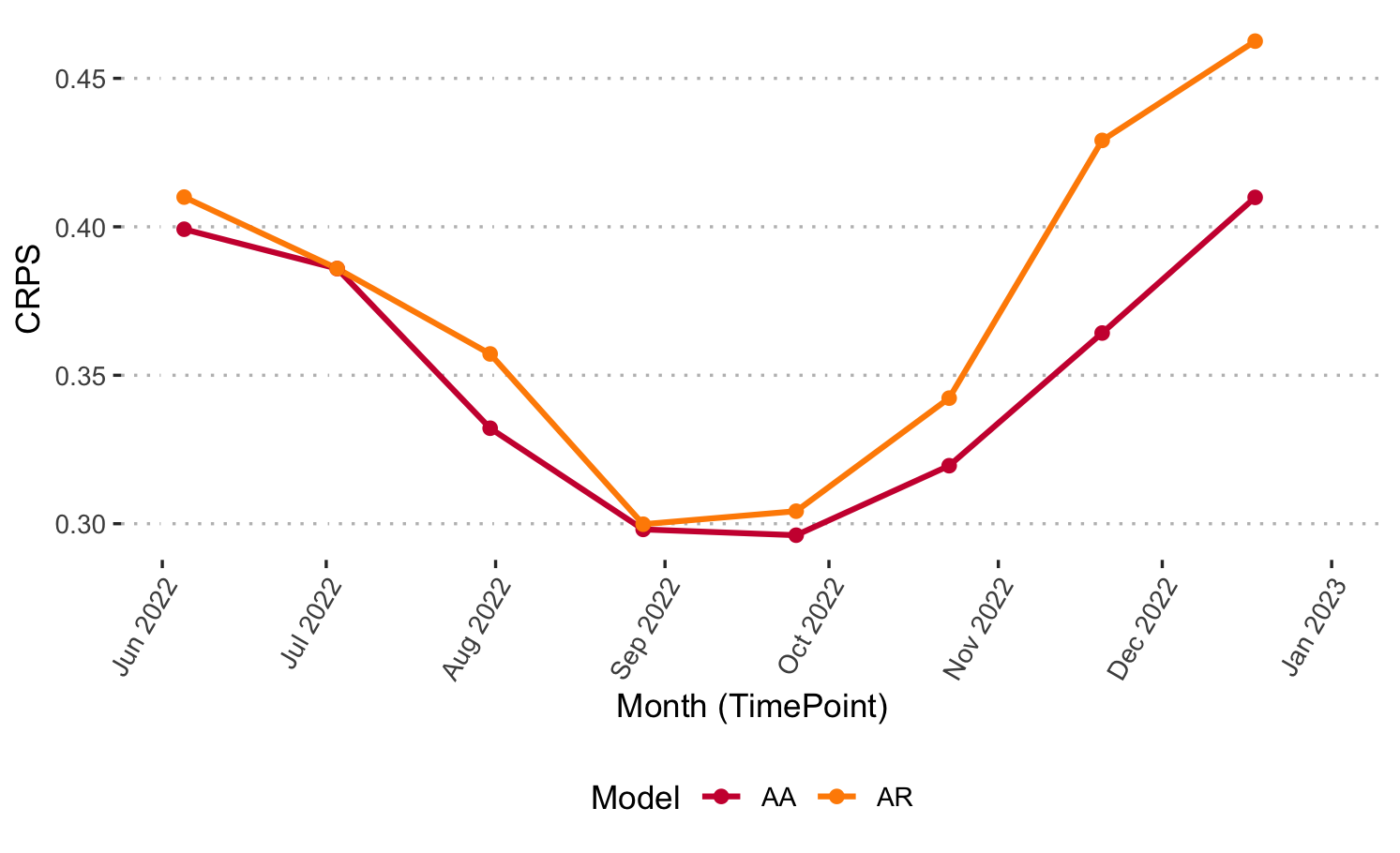

Supplement: S5 Fig — Figure displays the CRPS values for the amino acid (AA) distance informed model (red line) and the baseline autoregressive (AR) model (orange) over the eight-month prediction and validation period. Lower CRPS values indicate better performance. (TIFF) [file pcbi.1014329.s005.tiff]

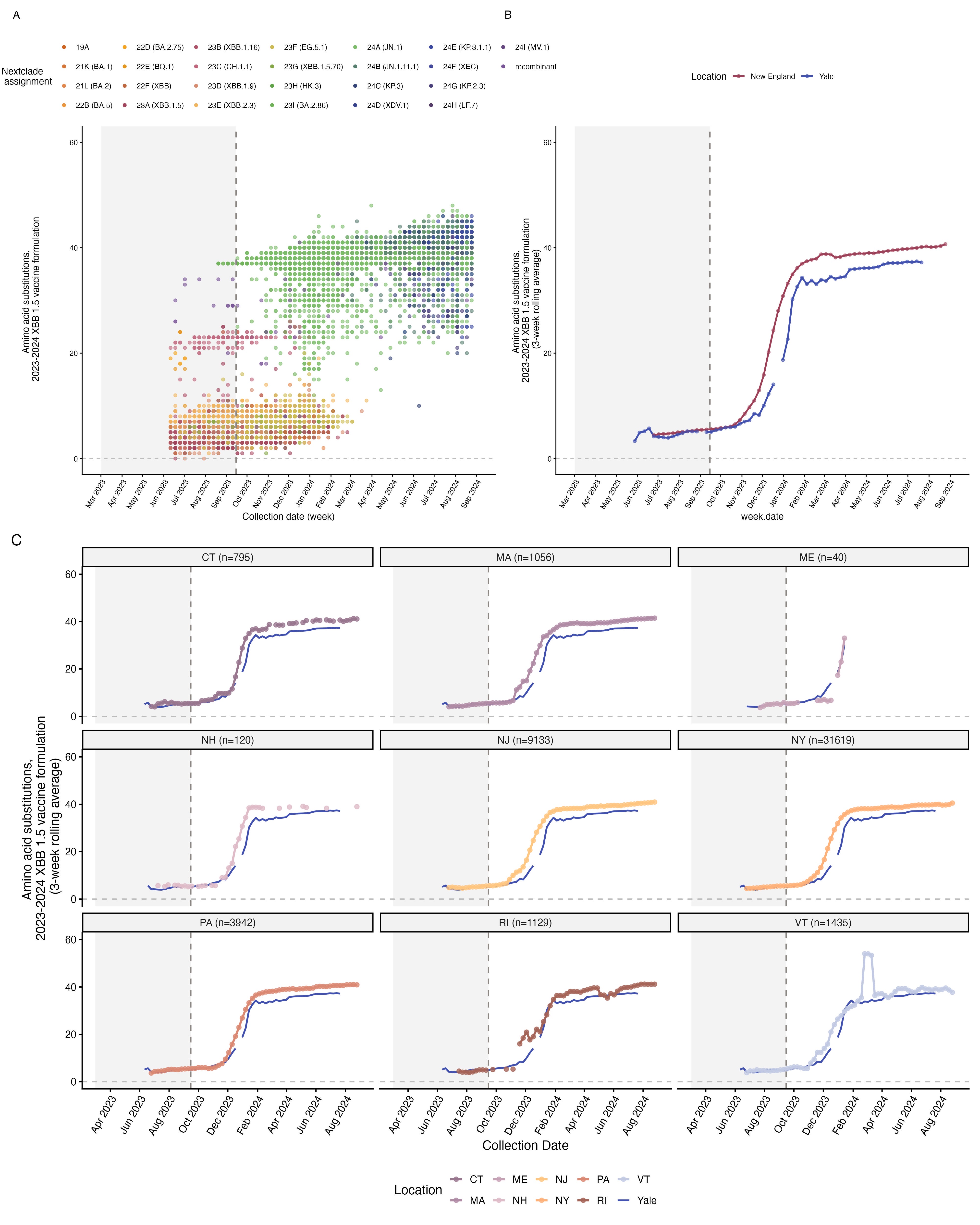

Supplement: S6 Fig — 1.5 formulation of mRNA COVID-19 vaccines. Sequences are aggregated by week from Jun 15, 2023, to August 30, 2024. Each dot represents a single sequenced case. The grey dotted line represents the introduction of the XBB.1.5. vaccine update. The grey shaded area represents sequences that were collected prior to XBB.1.5 vaccine availability. (A) All northeast sequences combined and colored by Nextclade lineage assignment. (B) Comparison of mean amino acid substitutions over time between Northeast sequences (red) versus Connecticut (blue) sequences. (C) State-level comparisons, where sequences from each of the Northeast states are plotted against Connecticut (blue) sequences to highlight local variations in the timing and magnitude of amino acid substitutions. (JPG) [file pcbi.1014329.s006.jpg]

**Supplementary Methods**


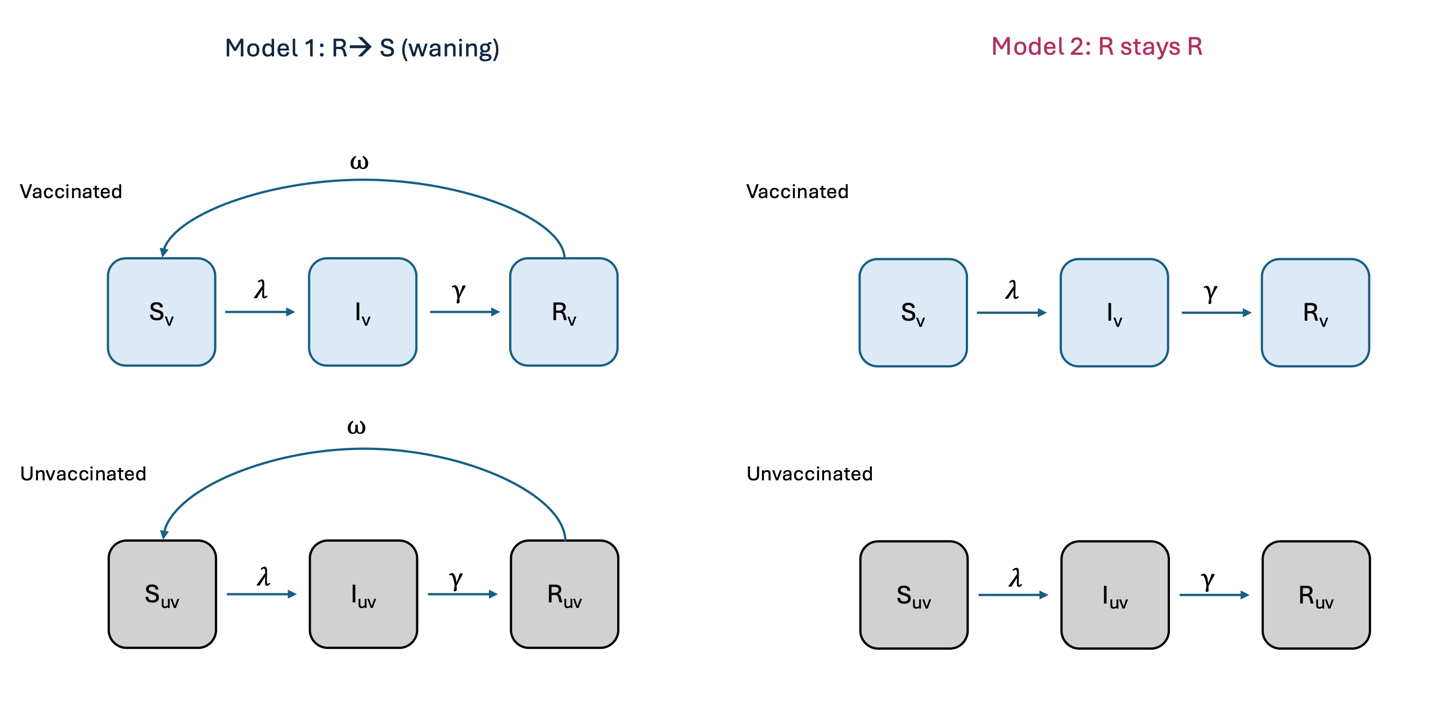


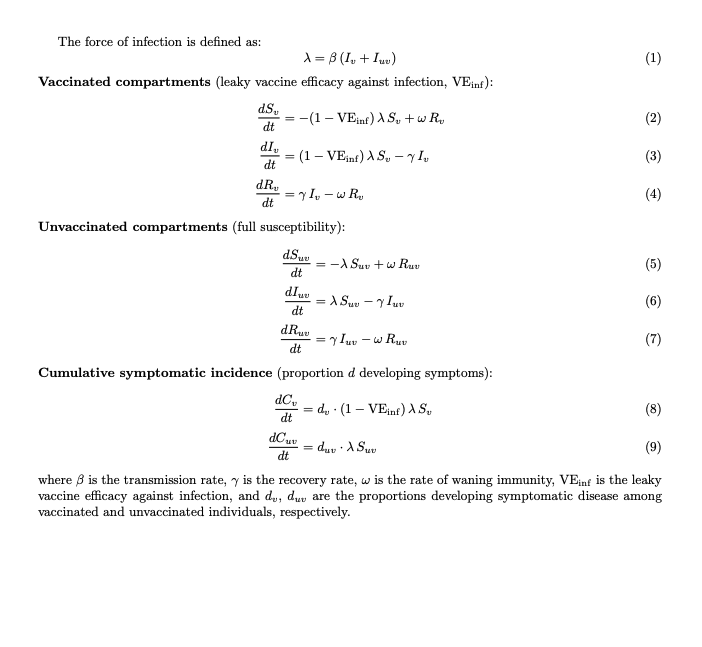

Supplement: S1 File — (DOCX) [file pcbi.1014329.s008.docx]
