# Supplementary material for: Utilizing virus genomic surveillance to predict vaccine effectiveness
Source: PLoS Comput Biol. 2026 May 26;22(5):e1014329. doi: 10.1371/journal.pcbi.1014329 (PMC13258143; doi:10.1371/journal.pcbi.1014329)
Supplement: S1 Table — (DOCX) [file pcbi.1014329.s007.docx]

**S1 Table. Fixed model parameter values.**

| **Description** | **Symbol** | **Values** | **Source** |
| --- | --- | --- | --- |
| Vaccination coverage | $\alpha$ | 0.75 | Assumed |
| VE against infection (leaky, primary vaccination series against Delta) | VE_inf | 0.71 | (*1*) |
| VE against symptomatic disease given infection (primary vax vs delta ) | VE_sym | 0.80 | (*2*) |
| Rate of recovery from infection | γ | 1/7 day⁻¹ | Assumed |
| Rate of waning immunity (Model 1) | ω | 1/90 day⁻¹ | Assumed |
| Rate of waning immunity (Model 2) | ω | 0 | Assumed |
| Effective reproductive number (sensitivity range) | R_eff | 1.5, 2.0, 2.5 | Assumed |
| Proportion symptomatic given infection, unvaccinated | d_uv | 0.75 | Assumed |
| Proportion symptomatic given infection, vaccinated [d_uv × (1−VE_sym)] | d_v | 0.15 | Derived |
| Population size | N | 10,000 | Assumed |

References

1. S. P. Andeweg *et al.*, Protection of COVID-19 vaccination and previous infection against Omicron BA.1, BA.2 and Delta SARS-CoV-2 infections. *Nature Communications* **13**, 4738 (2022).

2. J. Lopez Bernal *et al.*, Effectiveness of Covid-19 Vaccines against the B.1.617.2 (Delta) Variant. *New England Journal of Medicine* **385**, 585-594 (2021).
