# Supplementary material for: Utilizing virus genomic surveillance to predict vaccine effectiveness
Source: PLoS Comput Biol. 2026 May 26;22(5):e1014329. doi: 10.1371/journal.pcbi.1014329 (PMC13258143; doi:10.1371/journal.pcbi.1014329)
Supplement: Attachment — Submitted filename: PCOMPBIOL-D-25-01498 05-Sep-25.pdf [file pcbi.1014329.s010.pdf]

“Utilizing virus genomic surveillance to predict vaccine effectiveness”

PLOS Computational Biology PCOMPBIOL-D-25-01498

Comments to the authors

Major concerns

This study utilizes a unique dataset in which genomic surveillance data from a hospital system were linked with clinical metadata (line 248); however, using public data will be necessary if the VE model described here is eventually used at scale to inform vaccine update efforts. The authors should include an analysis of publicly available data for the USA or Northeast USA, time-matched to their test dataset. While preliminary, it would provide a rough idea of how the model would perform in a “real-world” scenario.

Minor concerns and suggested revisions

1. **Lines 141-153:** This paragraph states that Supplemental Figure 2 shows analysis with the full spike sequence. The caption of Supplemental Figure 2 states that it shows analysis with the RBD sequence only. The caption of Figure 2B states that it shows the full sequence. Which is correct?
2. **Line 162:** Please include the year (2022?) with “October”, since there are multiple years within the dataset.
3. **Figure 2B and Supplemental Figures 2-3:** These plots are a great way of collapsing lots of data into two dimensions; however, this reviewer struggled to understand what individual dots meant. Why are there multiple dots per variant in a given week? Does each dot mean “≥1 sequence of variant [color] was observed in week [x] with [y] substitutions”? If so, how can the reader tell if multiple variants (colors) were observed in a given week with the same number of substitutions? Additional explanation is needed in the figure captions.
4. **Figure 2C and Figure 3:** The reader can’t readily compare the observed % VE in Figure 2C with the predicted % *reduction* in VE in Figure 3. Adding a % VE reduction line or an additional panel to this figure would be immensely helpful.
5. **Figure 4 and Supplemental Figure 3:** These figures are highly complementary. I recommend merging them so that Figure 4 has two panels, rather than relegating one to Supplementary Materials.
6. **Line 452:** The provided GitHub repository (current commit: a91c067) does not contain any code or data as claimed in the data and materials availability. This must be updated before publication.
7. **Supplementary Materials line 198:** Please define CRPS.
8. **Supplementary Materials line 206:** Please provide an EPI_SET ID for the sequences used in the analysis.
