# Supplementary material for: Utilizing virus genomic surveillance to predict vaccine effectiveness
Source: PLoS Comput Biol. 2026 May 26;22(5):e1014329. doi: 10.1371/journal.pcbi.1014329 (PMC13258143; doi:10.1371/journal.pcbi.1014329)
Supplement: Attachment — Submitted filename: review_comments.pdf [file pcbi.1014329.s011.pdf]

It was with great enthusiasm that I read the paper by Kwon et al. The idea of using sequence distance (in this case, amino-acid distance within the spike protein) as a predictor of time-varying vaccine effectiveness is an appealing one, and I found the paper genuinely interesting and well-written.

I do, however, have a couple of concerns with the paper which prevent me from recommending publication in its current form.

Major concerns:

My main reservation about the paper concerns statistical significance. The authors do generally provide 95% CIs for their results, which is very helpful. However, in the majority of cases, those credible intervals reveal that the results are not statistically significant, at least if a $p=0.05$ definition of significance is used, as is implied by the CIs.

This begins already in the abstract, which says:

* *"Each 10 amino acid increase in distance in the spike gene corresponds to a predicted 15.4% (95% Credible intervals (CrI): –2.0%, 34.6%) reduction in VE"* (i.e. not significantly different from 0)

* *"For the 2023/24 updated vaccine, spike distance rose from 12.25 to 30.23, predicting a 43.4% (95% CrI: –5.7%, 90.1%) drop in VE"* (again, not significantly different from "no effect")

I find it somewhat problematic that the same abstract, only a few lines before, reads *"We found that spike gene amino acid distance* ***showed a strong negative correlation with VE*** *over time"* (emphasis mine). This certainly risks giving an impression of clearly significant results.
Similarly, the Discussion states *"We demonstrated a* ***clear association*** *between increasing amino acid distance […] and declining population-level VE"*.

This lack of significance is, from what I can tell, never discussed in the text. The word "significance" appears only once, to say *"we observed a significant increase in mean amino acid distance"*.

The significance problem continues throughout the text. For example, lines 162-163 read *"In early October, within three weeks of the bivalent booster introduction, mean VE increased by 12 percentage points to 40% (95% CrI: 6.22%, 63.19%), compared to 28% (95% CrI: -9.98%, 54.58%) in late August 2022."* In other words, while the October VE of 40% is significantly different from 0%, it is not significantly different from the 28% observed in August.

Thus, it seems to me that either the authors must introduce substantial caveats around the significance of the results, which will necessarily also affect the overall impact of the paper, or they will have to revisit the model formulation or significance calculations.
The reason that I mention the latter is that I am in fact surprised that so many of these results turn out to be statistically insignificant, when the signal – naively – appears very convincing. Just from looking at the figures, I would have guessed that a significant relationship exists.
Is the problem that the VE estimate is too uncertain? That the correlated random effects of the VE model (allowing associations between vaccination status and case/control probability to vary in time) simply allow for *too much* flexibility in fitting, leading to highly uncertain parameters?

The authors (to their credit) mention that they did not account for depletion of susceptibles, and specifically did not account for the possibility that this depletion would affect vaccinated and

unvaccinated populations differently. This seems to me a rather important point, and I think that the author should at the very least offer an analysis (or back-of-the-envelope calculation) which justifies neglecting this.

Furthermore, it does not seem that either the code or the data is available, despite this being stated in the Data and Code Availability statement. I looked through the GitHub repository and found that it contained, aside from the usual README and License documents, no actual code. It did contain an .Rproj file, but this did not contain the code or data either.

Minor points:
* I would like to see – just as a point of comparison – how the analysis would fare if the full (Levensthein) nucleotide distance were to be used, including ostensibly silent mutations, rather than the amino acid distance.
* Lines 149-151: I am not sure this within-group variation comparison is all that salient, for a couple of reasons. First, the relative variation within Delta is actually higher than for BA.1. Secondly, and more importantly, I am not sure it is very informative to report this as an average over the entire “run” of a given variant, since the main source of within-variant variation is the gradual accrual of random single point mutations in different (sub)lineages/chains of transmission. In other words, in order for the analysis to be useful, one should at least report a time-varying (coefficient of?) variation. Could be a supplementary figure.
* Lines 152-153: the phrasing makes it a bit unclear if it is the supplement, or the main text, that restricts the analysis to the RBD.
* Fig 2: There are gaps in the vaccination curve (panel A). I could not find that they were addressed anywhere, although I suspect I must have missed it? I assume insufficient data?
* Fig 3: Is there any particular reason that a similar analysis is not performed during other/longer stretches?
* Line 42: a comma before “aligned” seems misplaced.
