# Supplementary material for: Utilizing virus genomic surveillance to predict vaccine effectiveness
Source: PLoS Comput Biol. 2026 May 26;22(5):e1014329. doi: 10.1371/journal.pcbi.1014329 (PMC13258143; doi:10.1371/journal.pcbi.1014329)
Supplement: Attachment — Submitted filename: PCOMPBIOL-Response_Jan26.pdf [file pcbi.1014329.s013.pdf]

PCOMPBIOL-D-25-01498
Utilizing virus genomic surveillance to predict vaccine effectiveness
PLOS Computational Biology

Reviewer 1: It was with great enthusiasm that I read the paper by Kwon et al. The idea of using sequence distance (in this case, amino-acid distance within the spike protein) as a predictor of time-varying vaccine effectiveness is an appealing one, and I found the paper genuinely interesting and well-written. I do, however, have a couple of concerns with the paper which prevent me from recommending publication in its current form.

RESPONSE: We thank the reviewer for their positive assessment and address their specific comments in blue text below.

Major concerns:

My main reservation about the paper concerns statistical significance. The authors do generally provide 95% CIs for their results, which is very helpful. However, in the majority of cases, those credible intervals reveal that the results are not statistically significant, at least if a p=0.05 definition of significance is used, as is implied by the CIs.

This begins already in the abstract, which says:

* "Each 10 amino acid increase in distance in the spike gene corresponds to a predicted 15.4% (95% Credible intervals (CrI): –2.0%, 34.6%) reduction in VE" (i.e. not significantly different from 0)

* "For the 2023/24 updated vaccine, spike distance rose from 12.25 to 30.23, predicting a 43.4% (95% CrI: –5.7%, 90.1%) drop in VE" (again, not significantly different from "no effect")

I find it somewhat problematic that the same abstract, only a few lines before, reads "We found that spike gene amino acid distance showed a strong negative correlation with VE over time" (emphasis mine). This certainly risks giving an impression of clearly significant results.

Similarly, the Discussion states "We demonstrated a clear association between increasing amino acid distance [...] and declining population-level VE".

This lack of significance is, from what I can tell, never discussed in the text. The word "significance" appears only once, to say "we observed a significant increase in mean amino acid distance".

The significance problem continues throughout the text. For example, lines 162-163 read "In early October, within three weeks of the bivalent booster introduction, mean VE increased by 12 percentage points to 40% (95% CrI: 6.22%, 63.19%), compared to 28% (95% CrI: -9.98%, 54.58%) in late August 2022." In other words, while the October VE of 40% is significantly different from 0%, it is not significantly different from the 28% observed in August.

Thus, it seems to me that either the authors must introduce substantial caveats around the significance of the results, which will necessarily also affect the overall impact of the paper, or they will have to revisit the model formulation or significance calculations.

The reason that I mention the latter is that I am in fact surprised that so many of these results turn out to be statistically insignificant, when the signal – naively – appears very convincing. Just from looking at the figures, I would have guessed that a significant relationship exists. Is the problem that the VE estimate is too uncertain? That the correlated random effects of the VE model (allowing associations between vaccination status and case/control probability to vary in time) simply allow for too much flexibility in fitting, leading to highly uncertain parameters?

RESPONSE: Thank you very much for your thorough reading of our manuscript. We would like to clarify our methodological philosophy and the modeling choices that led to these results:

1. The second-stage meta regression model: The concerns regarding statistical significance primarily relate to the parameter $\boldsymbol{\gamma_1}$ in our second-stage meta-regression model (Line 489 Methods).

   In our study, we estimate the relationship between changes in vaccine effectiveness (VE) and amino acid (AA) in two distinct stages:
    - Stage 1: Using case-control data alone (without genomic information), we use a time-varying framework to obtain week-level estimates of vaccine effectiveness (VE_t) and their associated errors ($\hat{\sigma}_t{}^2$) (Line 432 Methods).
    - Stage 2: We incorporate genomic information in the form of AA. We quantify the relationship between the AA and VE_t obtained from the first stage using the meta-regression model (Line 489 Methods), which can also be written as:

$$\hat{\beta}_{2t} = \gamma_0 + \boldsymbol{\gamma_1} AA_t + \alpha_t + \varepsilon_t + \theta_t$$

where $\theta_t \sim \mathrm{N}\left(0,\ \hat{\sigma}_t{}^2\right)$ represents the propagated uncertainty from the first stage.

2. Wide credible interval: The main reason our credible intervals appear wider than what the "naïve" visual signal might suggest is due to our commitment to rigorous statistical inference in a two-stage model. We ensure that the uncertainty ($\hat{\sigma}_t{}^2$) from the first stage is fully incorporated into the second stage.

   As you correctly intuited, if we had removed θ_t and assumed $\hat{\beta}_{2t} = \gamma_0 + \boldsymbol{\gamma_1} AA_t + \alpha_t + \varepsilon_t$ (treating VE from stage 1 as a fixed point without error, $\hat{\sigma}_t{}^2 = 0$), our results for $\boldsymbol{\gamma_1}$ would likely be "significant" by standard definitions. However, our approach prioritizes statistical rigor when implementing a two-stage model to avoid type 1 errors and spurious correlations.

3. Results interpretation: While the current 95% credible intervals may overlap the null, the posterior distribution suggests that the negative association between amino acid distance and VE remains strong. For instance, the relationship is "significant" at an alpha level of 0.1, with a 90% credible interval [1.6, 32.35]. We believe that reporting the full uncertainty – rather than emphasizing statistical "significance" – provides a more transparent view of the evidence.

4. Manuscript revisions: We have added a paragraph to the Discussion explicitly explaining the nuances of the width of our credible intervals

   (Discussion section lines 335 – 339; Added texts are in bold)
   *Lastly, we implemented a two-stage approximation to a full hierarchical Bayesian model****. While*** *this remains an approximation****, w****e addressed first-stage uncertainty through a meta-regression framework with autoregressive random effects****. Although this approach likely resulted in wider credible intervals, it prevented the model from overestimating precision and minimized the risk of Type I errors.***

The authors (to their credit) mention that they did not account for depletion of susceptibles, and specifically did not account for the possibility that this depletion would affect vaccinated and unvaccinated populations differently. This seems to me a rather important point, and I think that the author should at the very least offer an analysis (or back-of-the-envelope calculation) which justifies neglecting this.

RESPONSE: We thank the reviewer for highlighting the potential for bias from the differential depletion of susceptibles; this comment has given us an opportunity to elaborate on this important nuance within the manuscript. Rather than performing a "back-of-the-envelope" calculation, we have opted to provide a qualitative assessment of the potential magnitude of this bias based on an established simulation framework for pathogens with SARS-CoV-2-like characteristics. We believe this is a more robust approach, as it avoids making several unverifiable assumptions. The literature indicates that the magnitude of this bias is non-monotonic and highly dependent on the interplay of initial VE, true waning rates, and outbreak timing (Kahn et al., 2023; PMID: 37771045).

Specific to our study, our assessment suggests that the magnitude of this bias is primarily mitigated by focusing our primary analysis to recently vaccinated individuals (14-90 days since their last dose). Kahn et al. demonstrate that "spurious waning" scales with time since vaccination; the expected magnitude of this dynamic across various assumptions can be visualized in Panels A-C of Figure 1 in their study. By focusing on the 14–90 day window on the x-axis, one can see that we effectively estimate the vaccine effectiveness before differential depletion reaches a magnitude sufficient to significantly skew the results.

Furthermore, the high reinfection rates characteristic of the Omicron era provides a natural buffer against sustained and monotonic depletion bias. Due to Omicron's ability to escape previous immunity, individuals with prior immunity frequently re-enter the susceptible pool. This "circular" flow of susceptibility causes the unvaccinated pool to effectively "refill", ultimately mitigating the differential depletion that traditionally drives spurious waning over time. While formal methods to quantify this magnitude across emerging variants remain an important future direction, we believe our current methodological safeguards potentially minimize the risk of significant bias in our estimates.

References:

Andrejko et al. 2023 (PMID: 36702469)

Kahn et al. 2022 (PMID: 35081612)

Kahn et al. 2023 (PMID: 37771045)

We have expanded the **Discussion** (Lines 328–334) to address the nuances of this potential bias:

Additionally, we did not account for potential bias related to the depletion of susceptible people, which can occur when natural immunity from infection is acquired faster in the unvaccinated group than in the vaccinated group, potentially underestimating VE and leading to incorrect conclusions about time-varying effectiveness(38, 39). ***We mitigated this bias by focusing our primary analysis on recently vaccinated individuals (14-90 days), thereby capturing VE estimates before differential depletion could significantly skew the results (40). Furthermore, the high reinfection rates of emerging variants provide a natural buffer against sustained depletion bias(40). Because immune escape variants allow individuals with previous infection-induced immunity to re-enter the susceptible pool, the resulting "refilling" of the unvaccinated group further reduces statistical artifacts associated with spurious waning (40).***

Furthermore, it does not seem that either the code or the data is available, despite this being stated in the Data and Code Availability statement. I looked through the GitHub repository and found that it contained, aside from the usual README and License documents, no actual code. It did contain an .Rproj file, but this did not contain the code or data either.

RESPONSE: Thank you for catching that. This is now updated, and the link is here: https://github.com/jiyekwon/Yale_VE_CompBio

Minor points:

* I would like to see – just as a point of comparison – how the analysis would fare if the full (Levensthein) nucleotide distance were to be used, including ostensibly silent mutations, rather than the amino acid distance.

RESPONSE: We recognize that evaluating the full nucleotide distance, including ostensibly silent mutations, provides an important point of comparison for our amino-acid-based approach. The specific nucleotide sequences of the vaccines are not publicly available; we conducted this comparative analysis by calculating the nucleotide distance relative to the reference sequence used for the original vaccine formulation. We have incorporated this analysis as an updated supplementary figure (**Supplemental Figure S3; Panels C & D**), which demonstrates a strong correlation between nucleotide and amino acid distance (Spearman correlation coefficient R^2 = 0.902). We agree that nucleotide distance may be highly useful for adapting this framework to other pathogens or vaccine platforms. In the specific context of SARS-CoV-2 mRNA vaccines, we believe that the amino-acid-level Spike

gene remains the most biologically relevant metric since AA substitutions in the Spike protein are the primary drivers of the phenotypic changes and protein-level immune escape that may impact population-level vaccine effectiveness.

Results lines 166 – 172

*We also tracked temporal changes in nucleotide differences relative to the NCBI reference sequence (**Supplemental Figure 3C**). Overall, we demonstrated high correlation between the spike gene amino acid differences used in our main analysis and two alternative measures: restricting the analysis to amino acid substitutions in the receptor binding domain alone (**Supplemental Figure 3B**) or extending the analysis to more granular, nucleotide changes within the spike region (**Supplemental Figure 3D**).*

* Lines 149-151: I am not sure this within-group variation comparison is all that salient, for a couple of reasons. First, the relative variation within Delta is actually higher than for BA.1. Secondly, and more importantly, I am not sure it is very informative to report this as an average over the entire "run" of a given variant, since the main source of within-variant variation is the gradual accrual of random single point mutations in different (sub)lineages/chains of transmission. In other words, in order for the analysis to be useful, one should at least report a time-varying (coefficient of?) variation. Could be a supplementary figure.

RESPONSE: We agree that reporting a static average over the entire duration of a variant may obscure the temporal dynamics of viral evolution, such as the gradual accrual of mutations within lineages over time. To address this, we have incorporated a new analysis visualizing the temporal trend of variance by utilizing a 30-day rolling standard deviation of amino acid substitutions, faceted by variant. This time-varying approach provides a more granular view of how within-group diversity evolved over the course of each variant's circulation. In the main text, we have removed the static averages (the 33.55 ± 1.25 part) in favor of the temporal analysis and added these results in **supplemental figure S2** (new figure).

Results lines 163-164:

*Within-group variations were also evident **both between variants and temporally throughout their respective periods of circulation (Supplemental Figure 2).***

* Lines 152-153: the phrasing makes it a bit unclear if it is the supplement, or the main text, that restricts the analysis to the RBD.

RESPONSE: We have revised the text to explicitly distinguish these two figures. The main text clarifies that Figure 2 utilizes the full spike gene, while the supplemental figures 3A & 3B relate to RBD-specific analysis. The revised manuscript (results lines 164-166) now reads "*Our analysis of the full spike gene captured a broader range of variations (**Figure 2**), offering a more comprehensive view than receptor binding domain alone (**Supplemental Figure 3A & 3B**).*"

* Fig 2: There are gaps in the vaccination curve (panel A). I could not find that they were addressed anywhere, although I suspect I must have missed it? I assume insufficient data?

RESPONSE: We thank the reviewer for careful reading and catching this detail. The gaps observed in Figure 2A correspond to specific weeks where there were zero observations for a given vaccination category (14-90 days) within the three-week rolling average window. We have added a clarifying note to the Figure 2 legend stating that these gaps reflect a lack of observations during those intervals.

Updated legend:

*Figure 2. Relationship between spike gene amino acid distance to original vaccine formulation and time-varying vaccine effectiveness for case sequences included in the model calibration, April 2021 to September 2022..... (A) Comparison of sequences between unvaccinated (yellow) and recently vaccinated individuals (red; 14-90 days since the most recent dose). **The red line represents the 3-week rolling average of (%) recently vaccinated individuals among sequenced infections over time; gaps in this line represent intervals with zero observations for this category.** ...*

* Fig 3: Is there any particular reason that a similar analysis is not performed during other/longer stretches?

RESPONSE: The primary objective of this specific analysis was to quantify the immediate impact of a new vaccine formulation on population-level effectiveness. We chose the analysis window based on the timing of the vaccine rollout and enforced symmetry in the months analyzed prior to and following the introduction to ensure a balanced comparison. While the four-month window pre- and post-rollout was selected as a practical period for observation, we also deliberately avoided extending the analysis too far out. The constant evolution of the virus would likely attenuate the direct signal of the vaccine update over a longer duration (e.g., true VE waning after update or the emergence of further distant variants). We believe this window provides the most focused view of the transition period.

We also recognize that those wishing to adopt this framework for other pathogens or vaccine platforms may modify these parameters based on their specific research context.

* Line 42: a comma before “aligned” seems misplaced.

RESPONSE: Thank you very much for catching that. We have deleted the misplaced comma in the manuscript.

Reviewer 2:

Comments to the authors

Major concerns

This study utilizes a unique dataset in which genomic surveillance data from a hospital system were linked with clinical metadata (line 248); however, using public data will be necessary if the VE model described here is eventually used at scale to inform vaccine update efforts. The authors should include an analysis of publicly available data for the USA or Northeast USA, time-matched to their test dataset. While preliminary, it would provide a rough idea of how the model would perform in a “real-world” scenario.

RESPONSE: We thank the reviewer for this excellent suggestion regarding the scalability of our framework. We agree that for this model to be adopted widely, it must demonstrate robust performance using publicly available genomic data. To address this, we conducted supplementary analysis using publicly available sequences from the NCBI database for the Northeast region of the United States, time-matched to the rollout of the XBB.1.5-adapted vaccine (June 2023 – August 2024; n = 49,266).

We compared the temporal trends in amino acid distance observed in our original dataset (Yale-CT) against these aggregated Northeast sequences. To ensure a granular comparison, we further stratified the public data by state, as an aggregated Northeast metric is often heavily weighted toward the high volume of sequences from New York. Our results, now included in **Supplemental Figure S5**, show that the general evolutionary trends observed in our clinical dataset are consistently reflected across the majority of the Northeast states. While we observed slight variations in the timing of distance increases, the overall trajectory of viral evolution relative to the vaccine formulation remained uniform. This comparison provides strong preliminary evidence that our model can effectively utilize public surveillance data to track the relationship between viral evolution and vaccine performance in real-world scenarios.

In the manuscript, we have added the following lines:

*Results (lines 240 -244): To assess the generalizability of our findings, we evaluated temporal changes in amino acid distance across the Northeast United States using all publicly available sequences from the NCBI database. Although the precise timing of the abrupt increase in mean amino acid distance varied by state, the overall evolutionary trajectory of circulating strains relative to the vaccine formulation closely mirrored our observations in Connecticut (**Supplemental Figure 5**).*

Minor concerns and suggested revisions

1. **Lines 141-153:** This paragraph states that Supplemental Figure 2 shows analysis with the full spike sequence. The caption of Supplemental Figure 2 states that it shows analysis with the RBD sequence only. The caption of Figure 2B states that it shows the full sequence. Which is correct?

RESPONSE: We have corrected the text and legends to ensure clarity. To clarify: **Figure 2B** utilizes the full spike sequence, while updated **Supplemental Figure 3**, panels A & B, provides the sensitivity analysis using the RBD sequence only. All captions and internal references have been updated to reflect this accurately.

The revised manuscript (results lines 164-166) now reads:

*Our analysis of the full spike gene captured a broader range of variations (**Figure 2**), offering a more comprehensive view than receptor binding domain alone (**Supplemental Figure 3A & 3B**).*

2. **Line 162:** Please include the year (2022?) with "October", since there are multiple years within the dataset.

RESPONSE: We have updated the text to include the year "2022" following "October".

3. **Figure 2B and Supplemental Figures 2-3:** These plots are a great way of collapsing lots of data into two dimensions; however, this reviewer struggled to understand what individual dots meant. Why are there multiple dots per variant in a given week? Does each dot mean "≥1 sequence of variant [color] was observed in week [x] with [y] substitutions"? If so, how can the reader tell if multiple variants (colors) were observed in a given week with the same number of substitutions? Additional explanation is needed in the figure captions.

RESPONSE: We appreciate reviewer's attention to the detail of the figures. We agree that the current figure legend is insufficient for understanding the individual data points.

Why are there multiple dots per variant in a given week?

- Since each dot is an individual sequence (sample), multiple dots appear in the same week. We have added this detail to the current version of the figure legend.

Does each dot mean "≥1 sequence of variant [color] was observed in week [x] with [y] substitutions"?

- Since each dot represents exactly one sequence, and the typical variant classification we know is assigned to each sampled case. In the case multiple variants co-circulate in the same week, we may see multiple colors showing up in the same week. And even within the same variant classification, multiple y-values may show up, due to within-variant heterogeneity.

We have revised the Figure 2 legend to explicitly state that each dot represents an individual sequenced case, along with other necessary clarifications:

Figure 2. Relationship between spike gene amino acid distance to original vaccine formulation and time-varying vaccine effectiveness for case sequences included in the model calibration, April 2021 to September 2022. *Panels (A) and (B) display individual dots, where each dot represents a single sequenced case included in the model calibration. The y-axis indicates the number of amino acid substitutions in the spike gene from the case sequences relative to the original formulation of mRNA COVID-19 vaccines. (A) Comparison of sequences between unvaccinated (yellow) and recently vaccinated individuals (red; 14-90 days since the most recent dose). The red line represents the 3-week rolling average of (%) recently vaccinated individuals among sequenced infections over time; gaps in this line represent intervals with zero observations for this category. (B) Sequences colored by variants of concern assigned to each case sequence (Alpha, black; Delta, blue; Omicron BA.1, red; BA.2, orange; BA.4, purple; BA.5, magenta; XBB.1.5, yellow). Vertical dispersion of dots of the same color reflects within-variant genetic heterogeneity, while the presence of multiple colors in a single week indicates variant co-circulation. (C) Week-level vaccine effectiveness (VE) estimates with 95% credible interval from the time-varying VE model for the period 14-90 days since the most recent dose. Vertical grey boxes denote distinct emergence periods, while the dotted line indicates the introduction of the updated bivalent vaccine formulation***.**

4. **Figure 2C and Figure 3:** The reader can't readily compare the observed % VE in Figure 2C with the predicted % *reduction* in VE in Figure 3. Adding a % VE reduction line or an additional panel to this figure would be immensely helpful.

RESPONSE: Following this suggestion, we have added a new panel A to Figure 3. This new panel overlays the observed time-varying VE estimates (Figure 2C; shown in green ribbon) with estimates from the second-stage amino-acid distance informed meta-regression model (purple ribbon). This comparison demonstrates that incorporating genetic distance information maintains alignment with observed trends while substantially narrowing the credible intervals.

5. **Figure 4 and Supplemental Figure 3:** These figures are highly complementary. I recommend merging them so that Figure 4 has two panels, rather than relegating one to Supplementary Materials.

RESPONSE: We have consolidated them into a single **Figure 4**, where **Figure 4A** now displays the content previously found in **Supplemental Figure 3**, and **Figure 4B** shows the original **Figure 4.** We agree that combining these figures enhances the manuscript's flow.

6. **Line 452:** The provided GitHub repository (current commit: a91c067) does not contain any code or data as claimed in the data and materials availability. This must be updated before publication.

RESPONSE: We apologize for the oversight regarding the GitHub repository contents at the time of initial submission. The GitHub repository has been updated and now contains all the necessary code and model scripts presented in this study.

7. **Supplementary Materials line 198:** Please define CRPS.

RESPONSE: Thank you for catching this omission. We have now defined the **Continuous Ranked Probability Score (CRPS)** upon its first mention in the Results (Line 205).

8. **Supplementary Materials line 206:** Please provide an EPI_SET ID for the sequences used in the analysis.

RESPONSE: Following the reviewer's suggestion, we have provided the full list of sequence identifiers. These can now be found in the updated GitHub repository at: Yale_VE_CompBio/data/yale_EPI.csv and as a supplementary data file.
