# Supplementary material for: Utilizing virus genomic surveillance to predict vaccine effectiveness
Source: PLoS Comput Biol. 2026 May 26;22(5):e1014329. doi: 10.1371/journal.pcbi.1014329 (PMC13258143; doi:10.1371/journal.pcbi.1014329)
Supplement: Attachment — Submitted filename: REV2_response_letter.pdf [file pcbi.1014329.s014.pdf]

Additional Editor Comments:

Thank you for your thorough response. Both Reviewers have recommended acceptance once the last comments from Reviewer #2 have been adequately addressed.
Thank you for overseeing the process for our manuscript. The reviewers' constructive feedback was helpful, and we are happy with the final version of the paper.

Reviewers' comments:

Reviewer's Responses to Questions

Comments to the Authors:
Please note here if the review is uploaded as an attachment.

Reviewer #1: Thank you for your thorough response to my review.

Reviewer #2: I find that the authors have adequately addressed my concerns. As such, I now recommend publication of the manuscript.

Even so, allow me to attach a few comments:

* Regarding the authors comment about gamma_1: it is plausible that gamma_1 is the main source of the uncertainty in question, however I am not sure if this comment was supposed to alleviate any concerns? gamma_1 is, in a sense, the most salient parameter.

* In the context of the potential impact on differential depletion of susceptibles, the authors write:
"Rather than performing a "back-of-the-envelope" calculation, we have opted to provide a qualitative assessment of the potential magnitude of this bias based on an established simulation framework for pathogens with SARS-CoV-2-like characteristics. We believe this is a more robust approach, as it avoids making several unverifiable assumptions."
This, I think, represents a bit of a misunderstanding. Broadly referring to a qualitative result does not involve fewer difficult assumptions than would an idealized model calculation. The difference being that, in the latter case, one could more easily state the assumptions. Stated assumptions are, on the whole, superior to implied ones.
For example, the authors write that, on the one hand "our assessment suggests that the magnitude of this bias is primarily mitigated by focusing our primary analysis to recently vaccinated individuals (14-90 days since their last dose)". On the other hand, they write "Furthermore, the high reinfection rates characteristic of the Omicron era provides [sic] a

natural buffer against sustained and monotonic depletion bias." However, it is altogether unclear whether the latter effect is of sufficient magnitude - on the timescale in question - to truly wash out the effects of differential depletion. I still believe the paper would benefit from a clearer treatment of this phenomenon, even if in a highly idealized model. However, take it as a suggestion.

RESPONSE: We appreciate the constructive feedback. To directly address the concern regarding the magnitude and timescale of the depletion of susceptibles bias, we developed a simple compartmental SIRS model simulation using parameters that resemble SARS-CoV-2-like characteristics.

We compare two scenarios:

1. Model 1 (SIRS / refilling pool): Recovered individuals from both vaccinated and unvaccinated groups re-enter susceptible pool (waning immunity: 90 days). This model reflects the high reinfection rates characteristic of the Omicron era. Notably, the observed VE initially dips below the true VE (~75%) as differential depletion begins but recovers toward the true VE (~90%) as the susceptible pools are progressively replenished. This non-monotonic behavior demonstrates that the refilling mechanism effectively attenuates the bias over the course of the epidemic.
2. Model 2 (SIR/ Sustained depletion): This model assumes that recovered individuals remain immune. In the absence of pool refilling, the bias is sustained and monotonically increasing — observed VE progressively diverges from the true VE as unvaccinated susceptibles are preferentially exhausted.

Our results demonstrate that across a range of transmission intensities (R_eff sensitivity analysis), Model 1 consistently yields VE estimates that track closer to the True VE. In contrast, Model 2 leads to a progressive underestimation of VE as the susceptible denominator is artificially exhausted.

**Description**	**Symbol**	**Values**	**Source**
Vaccination coverage	α	0.75	Assumed
VE against infection (leaky, primary vaccination series against Delta)	VE_inf	0.71	(*1*)
VE against symptomatic disease given infection (primary vax vs delta)	VE_sym	0.80	(*2*)
Rate of recovery from infection	γ	1/7 day^{-1}	Assumed

Rate of waning immunity (Model 1)	ω	1/90 day^{-1}	Assumed
Rate of waning immunity (Model 2)	ω	0	Assumed
Effective reproductive number (sensitivity range)	R_eff	1.5, 2.0, 2.5	Assumed
Proportion symptomatic given infection, unvaccinated	d_uv	0.75	Assumed
Proportion symptomatic given infection, vaccinated [d_uv × (1−VE_sym)]	d_v	0.15	Derived
Population size	N	10,000	Assumed

1. S. P. Andeweg *et al.*, Protection of COVID-19 vaccination and previous infection against Omicron BA.1, BA.2 and Delta SARS-CoV-2 infections. *Nature Communications* **13**, 4738 (2022).
2. J. Lopez Bernal *et al.*, Effectiveness of Covid-19 Vaccines against the B.1.617.2 (Delta) Variant. *New England Journal of Medicine* **385**, 585-594 (2021).

Model diagram and equations

Model 1: R→ S (waning)

Model 2: R stays R

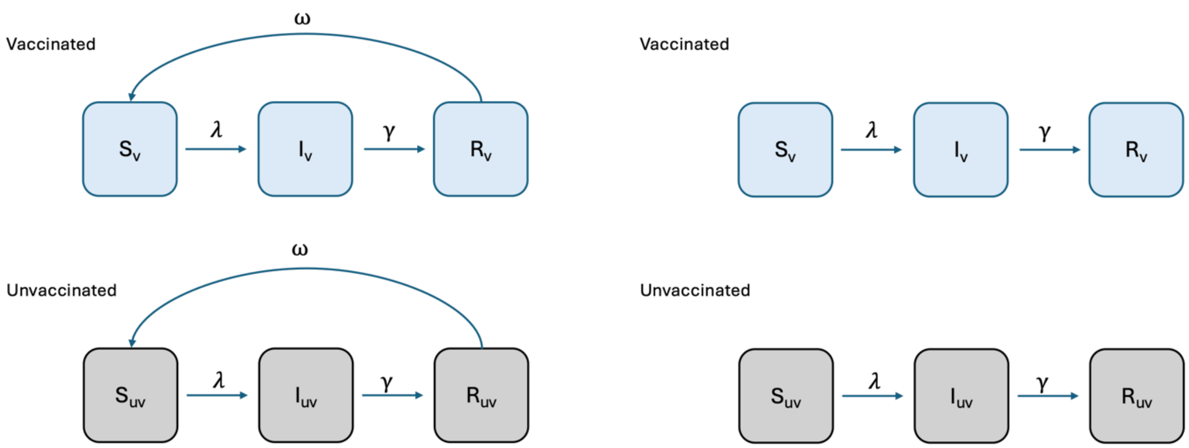

The force of infection is defined as:

$$\lambda = \beta\,(I_v + I_{uv}) \tag{1}$$

Vaccinated compartments (leaky vaccine efficacy against infection, $\mathrm{VE}_{\mathrm{inf}}$):

$$\frac{dS_v}{dt} = -(1 - \mathrm{VE}_{\mathrm{inf}})\,\lambda\,S_v + \omega\,R_v \tag{2}$$

$$\frac{dI_v}{dt} = (1 - \mathrm{VE}_{\mathrm{inf}})\,\lambda\,S_v - \gamma\,I_v \tag{3}$$

$$\frac{dR_v}{dt} = \gamma\,I_v - \omega\,R_v \tag{4}$$

Unvaccinated compartments (full susceptibility):

$$\frac{dS_{uv}}{dt} = -\lambda\,S_{uv} + \omega\,R_{uv} \tag{5}$$

$$\frac{dI_{uv}}{dt} = \lambda\,S_{uv} - \gamma\,I_{uv} \tag{6}$$

$$\frac{dR_{uv}}{dt} = \gamma\,I_{uv} - \omega\,R_{uv} \tag{7}$$

Cumulative symptomatic incidence (proportion d developing symptoms):

$$\frac{dC_v}{dt} = d_v \cdot (1 - \mathrm{VE}_{\mathrm{inf}})\,\lambda\,S_v \tag{8}$$

$$\frac{dC_{uv}}{dt} = d_{uv} \cdot \lambda\,S_{uv} \tag{9}$$

where β is the transmission rate, γ is the recovery rate, ω is the rate of waning immunity, $\mathrm{VE}_{\mathrm{inf}}$ is the leaky vaccine efficacy against infection, and d_v, d_{uv} are the proportions developing symptomatic disease among vaccinated and unvaccinated individuals, respectively.

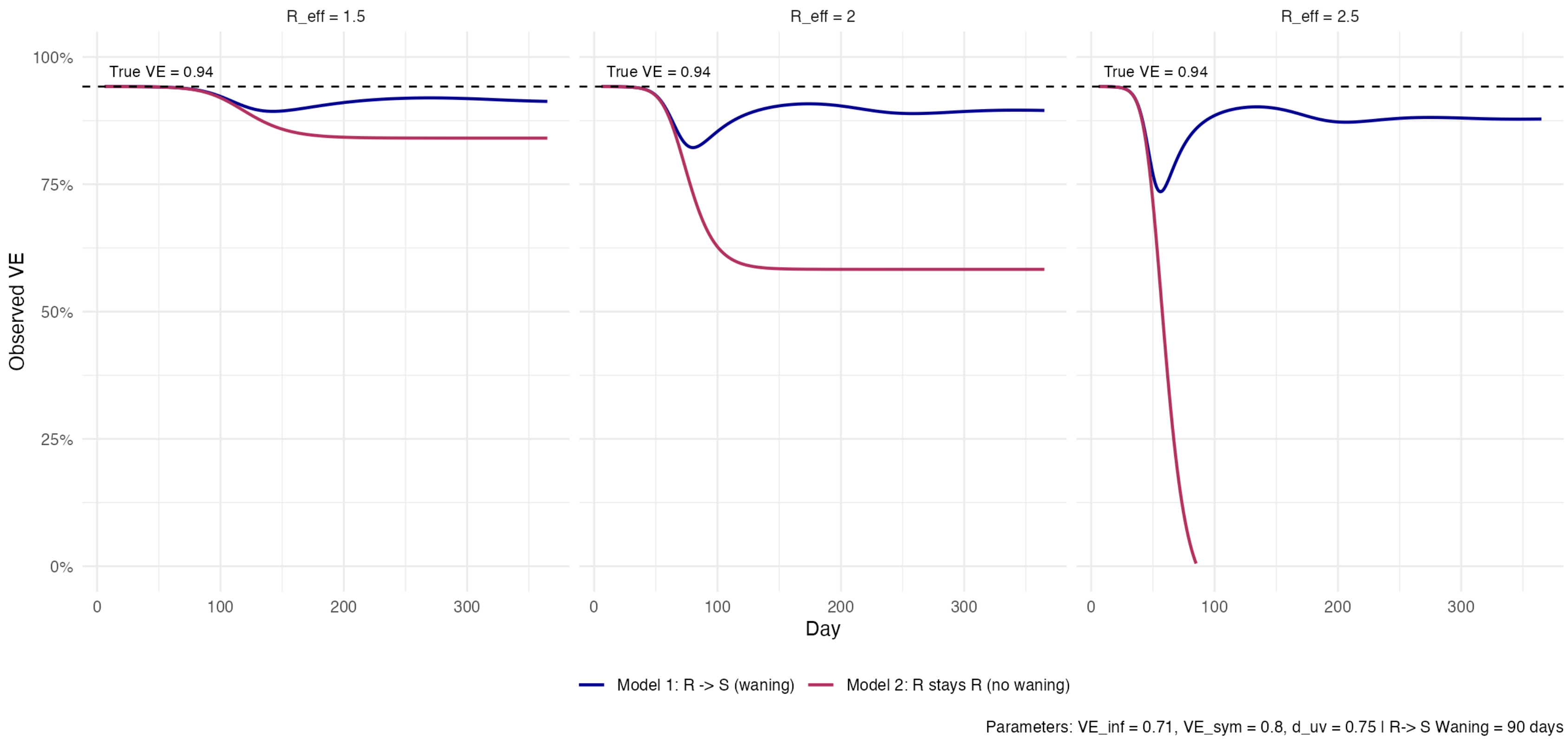
R_eff = 1.5
R_eff = 2
R_eff = 2.5
True VE = 0.94
100%
75%
50%
25%
0%
Observed VE
0
100
200
300
Day
Model 1: R -> S (waning)
Model 2: R stays R (no waning)
Parameters: VE_inf = 0.71, VE_sym = 0.8, d_uv = 0.75 | R-> S Waning = 90 days
